# Mode of action of elasnin as biofilm formation eradicator of methicillin-resistant *Staphylococcus aureus*

**DOI:** 10.3389/fmicb.2022.967845

**Published:** 2022-08-08

**Authors:** Lexin Long, Jordy Evan Sulaiman, Yao Xiao, Aifang Cheng, Ruojun Wang, Jessie James Malit, Wai Chuen Wong, Wenchao Liu, Yong-Xin Li, Feng Chen, Henry Lam, Pei-Yuan Qian

**Affiliations:** ^1^Department of Ocean Science, The Hong Kong University of Science and Technology, Kowloon, Hong Kong, China; ^2^SZU-HKUST Joint PhD Program in Marine Environmental Science, Shenzhen University, Shenzhen, China; ^3^Southern Marine Science and Engineering Guangdong Laboratory (Guangzhou), Guangzhou, China; ^4^Department of Chemical and Biological Engineering, The Hong Kong University of Science and Technology, Kowloon, Hong Kong SAR, China; ^5^Department of Chemistry, The University of Hong Kong, Pokfulam, Hong Kong SAR, China; ^6^The Swire Institute of Marine Science and Hong Kong Branch of Southern Marine Science and Engineering Guangdong Laboratory (Guangzhou), The University of Hong Kong, Pokfulam, Hong Kong SAR, China; ^7^Institute for Advanced Study, Shenzhen University, Shenzhen, China

**Keywords:** elasnin, biofilms, MRSA, resistance, antimicrobials, eradication, antibiofilm

## Abstract

Biofilm is made up of microbes and their extracellular matrix, making microorganisms highly tolerant, resistant, and resilient to a wide range of antimicrobials. Biofilm treatment with conventional antimicrobial agents can accelerate the evolution and spread of resistance due to the reduced efficacy and increased gene transfer and differentiation within biofilms. Therefore, effective biofilm-targeting compounds are currently highly sought after. In the present study, we identified elasnin as a potent biofilm-targeting compound against methicillin-resistant *Staphylococcus aureus* (MRSA). Elasnin effectively inhibited biofilm formation and especially eradicated the pre-formed biofilms of MRSA with low cytotoxicity and low risk of resistance development and retains its activity in a chronic wound biofilms model. A comprehensive mechanistic study using multi-omics and confocal and scanning electron microscopy revealed that elasnin induced the biofilm matrix destruction in a time-dependent manner and interfered with the cell division during the exponential phase, primarily by repressing the expression of virulence factors. Cells released from the elasnin-treated biofilms exhibited a defective appearance and became more sensitive to beta-lactam antibiotic penicillin G. Through gene overexpression and deletion assay, we discovered the key role of *sarZ* during elasnin-induced biofilm eradication. Overall, the present study identified elasnin as a potent biofilm eradicator against MRSA that harbors potential to be developed for biofilm removal and chronic wound treatment, and provided new insights into the molecular targets for biofilm eradication in MRSA.

## Introduction

Biofilms consist of microorganisms that grow on various surfaces. Microbial cells in biofilms are organised and embedded in a matrix that contains diverse self-produced extracellular polymeric substances (EPSs) including polysaccharides, proteins, nucleic acids and lipids (Flemming and Wingender, 2010; Flemming et al., 2016). Cells in biofilms are usually more tolerant, resistant, and resilient to external threats than their planktonic cells (Mah and O'Toole, 2001; Stewart and William Costerton, 2001; Hall and Mah, 2017). Consequently, conventional antimicrobial agents gradually lose their efficacies against biofilms, and extremely high concentrations are often required to eradicate the pre-formed biofilms (Hengzhuang et al., 2012; Wu et al., 2015).

Biofilm-associated infection is currently a major problem in clinics and the healthcare industry, accounting for about 80% of bacterial infections and 65% of nosocomial infections (Jamal et al., 2018). Biofilm formation is crucial for bacterial pathogenesis and is the leading cause of chronic and device-related infections (Madalina Mihai et al., 2015). Bacteria in the genus of Staphylococci are the most frequently reported source of biofilm-related infections. Among the reported cases of infections, one of the most dangerous pathogens in clinics to date is Staphylococcus aureus, whose resistance is related to biofilm formation (Lister and Horswill, 2014; Moormeier and Bayles, 2017). The typical process of Staphylococcus biofilm development requires the participation of many virulence factors and secreted substances such as adhesive surface proteins, degradative enzymes, EPSs and toxins (Antunes and Ferreira, 2011; Otto, 2019). These substances facilitate the adhesion and colonisation of bacteria and assist biofilm maturation and rapid cell proliferation. However, the dependence of matrix components on biofilm development for different *S. aureus* strains varies. For instance, PIA production encoded by ica operon is essential for methicillin-sensitive *S. aureus* (MSSA; O’Neill et al., 2007), whereas biofilms of methicillin-resistant *S. aureus* (MRSA) commonly form in an ica-independent manner and require adhesive surface proteins such as FnBPA and FnBPB (O’Neill et al., 2008), SasG (Corrigan et al., 2007), and Atl and extracellular DNA (eDNA; Houston et al., 2011; Bose et al., 2012). Apart from shaping the biofilm structure, degradative enzymes induce biofilm detachment and then facilitate the systemic dissemination of bacterial infection (Otto, 2019).

Biofilm formation is tightly controlled by the coordination of multiple signalling pathways, in which the quorum-sensing system Agr and the global regulators *SarA* protein family play the central roles (Jenul and Horswill, 2019; Otto, 2019). The Agr system encodes two different transcripts, namely, RNAII and RNAIII, in which RNAIII functions as an intracellular effector that directly or indirectly controls the expression of numerous virulence factors (e.g., proteases and surface adhesins), which are involved in biofilm formation, and cell wall hydrolases with roles in cell cycle and pathogenesis (Boisset et al., 2007; Monteiro et al., 2015). The *SarA* protein family consists of many members, such as *SarZ*, *SarX*, and *SarR*, most of which control the expression of virulence factors. Among all the proteins in this family, *SarA* is the most extensively studied. *SarA* is a global regulator that positively controls PIA synthesis, agr system, adhesins and toxins, and it represses its own expression and the production of proteases (Beenken et al., 2003; Cheung et al., 2008). Notably, many studies have revealed the interconnected roles of *sarA* and agr in the switching between the formation and detachment of *S. aureus* biofilm. The upregulation of adhesins, PIA, and protease inhibitors by *sarA* induces early biofilm adherence, and the activation of peptides and nucleases by agr assist biofilm dispersion (Beenken et al., 2010; Vasudevan, 2019).

Biofilm-related infections have significantly threatened human health. However, current biofilm control strategies are limited, and long-term and high-dose combinational treatments are general therapeutic strategies for biofilm infections (Wu et al., 2015). Previous drug discoveries mostly focused on treating planktonic cells and thus, cells in biofilms that had already been exposed to conventional antimicrobials are easier to develop resistance due to the limited penetration, enzyme degradation, increased gene transfer, and differentiation within biofilms (Mah and O’Toole, 2001). Recently, there are efforts in discovering new antibiofilm agents, such as those that target the EPS by inhibiting EPS production, binding to EPS adhesins or degrading the EPSs (dispersin B and DNase I). Other strategies such as inducing biofilm dispersal and metabolic interference are also potential directions for future drug discoveries and developments (Koo et al., 2017; Li and Lee, 2017).

In the present study, we showed that elasnin serves as a potent biofilm-targeting compound against MRSA which effectively inhibited and especially eradicated their pre-formed biofilms. Elasnin is a small molecule containing a 2-pyrone (α-pyrone) structure and was discovered to be effective in inhibiting marine biofilms in our previous study (Long et al., 2021). To elucidate elasnin’s mode of action, the combination of multi-omics analyses, microscopy imaging, gene manipulation, and other bioassays were performed. The results provided the detailed process of elasnin-induced biofilm eradication and highlighted the key genes that govern this process, including the transcriptional regulator gene *sarZ*.

## Materials and methods

### Strains, media and chemicals

Twelve actinobacterial strains (Supplementary Table S1) were obtained from the German Collection of Microorganisms and Cell Cultures (Braunschweig, Germany). The MRSA ATCC 43300, *Escherichia coli* ATCC 25922 and *S. aureus* ATCC 25923 strains were obtained from American Type Culture Collection. Soybean powder was obtained from Wugumf, Shenzhen, China. Soluble starch was obtained from Affymetrix, Santa Clara, CA, United States. Magnesium sulphate hydrate was obtained from Riedel-de-Haën, Seelze, Germany. Bacteriological peptone and tryptone soya broth (TSB) were obtained from Oxoid, Milan, Italy. Mueller-Hinton broth (MHB) was obtained from Fluka Chemie AG, Buchs, Switzerland. Proteinase K was obtained from Qiagen NV, Venlo, Netherlands. Phosphate-buffered saline (PBS) and DNase I were obtained from Thermo Fisher Scientific Inc., San Jose, CA, United States. Lysogeny broth (LB), glucose, 3-(4,5-dimethylthiazol-2-yl)-2,5-diphenyltetrazolium bromide (MTT) and 1-butanol were obtained from VWR International Ltd., Leicestershire, United Kingdom. Antibiotics, stains and all other chemicals were supplied by Sigma-Aldrich Corporation, Saint Louis, MO, United States.

### Antibacterial assay

Minimum inhibitory concentration (MIC) and minimum bactericidal concentration (MBC) were determined using MRSA ATCC 43300 and *E. coli* ATCC 25922 according to the Clinical and Laboratory Standards Institute guideline CLSI M100 (2018). In a typical procedure, a 10^5^ CFU/ml overnight culture of test strains was inoculated into MHB and treated with testing compounds in a series of concentrations. After incubation for 24 h, the minimum concentrations at which no bacterial growth was visible were recorded as the MICs. MBCs were measured following MIC assay by plating 1 ml of suitably diluted culture broth from each well on Mueller-Hinton agar (MHA) plate. MBC was defined as the lowest concentration at which an antimicrobial agent caused >99.9% reduction in cells. Each assay was performed in duplicate and repeated thrice.

MRSA ATCC 43300 was used for the concentration–response curve study. A culture of 4 × 10^5^ CFU/ml MRSA in the exponential phase was inoculated into MHB with various concentrations of elasnin and vancomycin in 15 ml falcon tubes. Tubes were incubated at 37°C on a rotary shaker for 24 h. Then, 1 ml of culture broth in each tube was diluted with MHB, and 1 ml of diluted bacteria was plated on MHA plates for CFU counting. Culture broth from each well was inoculated on two plates, and the experiments were repeated thrice.

### Antibiofilm assay

Minimum biofilm inhibitory concentration (MBIC) and minimal biofilm eradication concentration (MBEC) were determined as previously described (Nair et al., 2016; Yin et al., 2019; Long et al., 2021). The time-course biofilm formation on MRSA cells is shown in Supplementary Figure S1A. An overnight culture of test strains was diluted into approximately 10^7^ CFU/ml with LB and 0.5% glucose and treated with various concentrations of testing compounds in 96-well cell culture plates. These plates were then incubated at 37°C for 24 h and rinsed twice with 1 × PBS to remove non-adhering and planktonic cells. After rinsing, MTT staining assay was conducted to measure viable cells in the biofilms, because MTT can react with dehydrogenase enzymes in viable cells to form blue-violet formazan, which can be detected at 570 nm after dissolving in DMSO. MBIC_50_ and MBIC_90_ were defined as the lowest concentrations required to inhibit 50 and 90% of biofilm formation, respectively.

For MBEC assay, an overnight culture of test strains was incubated for 24 h in 96-well cell culture plates to form mature biofilm before rinsing twice with 1 × PBS and compound treatment. After 24 h of incubation at 37°C, each well was rinsed twice with 1 × PBS, and OD_570nm_ was recorded after MTT assay as described above. The lowest concentration of a compound resulting in 50% decrease in OD_570 nm_ were recorded as MBEC_50_. Biofilm inhibition/eradication efficiency was calculated using the following equation: Biofilm inhibition/eradication (%) = [1 − (OD_570nm_ of test compound) / (OD_570nm_ of control)] × 100%. Experiments were performed in triplicate and repeated thrice.

### Antibiofilm assays in modified Lubbock chronic wound biofilm model

The antibiofilm activity of elasnin was assessed using a modified LCWB model that simulates the conditions in chronic wounds (Sun et al., 2008; Brackman et al., 2011). Briefly, an overnight culture of MRSA was diluted into approximately 10^6^ CFU/ml with Bolton broth supplemented with 50% plasma (Sigma-Aldrich) and 5% freeze–thaw laked horse blood (Thermo Fisher Scientific). Then, the sample was treated with (MBIC) or without (MBEC) various concentrations of elasnin/vancomycin in 96-well cell culture plates. The plate was then incubated for 24 h at 37°C followed by medium removal and rinse with physiological saline (PS, Sigma-Aldrich). Afterwards, MTT staining was conducted to measure viable cells for MBIC assay. For MBEC assay, grown biofilm cells were treated with elasnin/vancomycin and incubated for another 24 h before MTT staining assay. Experiments were performed in triplicate and repeated twice, and MBIC and MBEC were calculated as described above.

### Monitoring of biofilm eradication and change in cell susceptibility

Mature biofilms of MRSA ATCC 43300 were first grown in 96-well cell culture plates and treated with various concentrations of elasnin as described above. Plates were then collected after 0, 3, 6, 12, 18, and 24 h of treatment, and OD570_nm_ values were recorded after rinsing and MTT assay. To assess the resistance development risks of elasnin, we conducted a susceptibility change study as previously described (Li et al., 2018). In a typical procedure, MRSA ATCC 43300 cells were grown in the presence of antibiotics (e.g., elasnin, vancomycin and ciprofloxacin) at final concentrations of 0.5×, 1×, 2×, 4×, and 8× of the MICs of the antibiotics. The cells were incubated at 37°C for 24 h, and we recorded the new MIC, which was the lowest concentration of antibiotic without no visible bacterial growth. Then, aliquots from the culture in which the second-highest antibiotic concentration (0.5 × of new MICs) showed visible growth were diluted for 1,000 times in MHB for the subsequent assay. The diluted culture was again grown in the presence of antibiotics at final concentrations of 0.5×, 1×, 2×, 4× and 8× of the previously measured MICs for 24 h, and the new MIC values were recorded. This process was repeated for 45 days. The experiment was performed in triplicate, and the fold change was calculated as the ratio between the measured MICs compared with the MIC on the first day.

### Cytotoxicity test

HT22 and Neuro2a (N2a) cells from ATCC were used in the MTT assay to test the cytotoxicity of the compounds. Cells were grown in DMEM with 10% FBS and 1% penicillin–streptomycin at 37°C with 5% CO_2_. Then, 5 × 10^3^ cells were seeded in each well of 96-well plates and cultured for 24 h. After cell treatment with different concentrations of the compounds dissolved in DMSO for another 24 h, 20 μl of MTT (5 mg/ml) was added to each well, followed by incubation for 4 h at 37°C before adding 100 μl of DMSO to dissolve formazan. The absorbance was measured using the Multiskan^™^ FC microplate photometer at 570 nm. IC_50_ data were analysed using the GraphPad Prism software.

### Confocal laser scanning microscopy observation with biofilm staining

Biofilms were grown on glass cover slides as described for the MBIC and MBEC assay. Treated biofilms were then rinsed twice with 1 × PBS and stained with FilmTracer^™^ FM^®^ 1–43 green biofilm cell stain and FilmTracer^™^ SYPRO^®^ Ruby Biofilm Matrix Stain at room temperature for 30 min in the dark. Leica Sp8 confocal microscope was used to observe the cells and the matrix in the biofilm at 488 nm.

To visualise the changes in biofilm matrix components after elasnin treatment, we prepared the biofilms as described above in the MBEC assays and stained them with TOTO^™^-1 Iodide and Concanavalin A to observe eDNA and polysaccharides within the biofilm matrix according to the manufacturer’s instruction. A Zeiss LSM 710 confocal microscope was used for observation, and ImageJ was used for quantification.

### Total RNA extraction and transcriptomic analysis

Overnight cultures of 10^7^ CFU/ml MRSA cells were inoculated into TSB complemented with 0.5% glucose (TSBG) at 37°C to obtain mature biofilms. After 24 h of incubation, mature biofilms were rinsed twice with 1 × PBS and treated with 5 μg/ml elasnin or media. Biofilm and released cells were collected at 6 and 12 h, and RNA was immediately stabilised with RNAprotect bacterial Reagent (Qiagen, Hilden, German) according to the manufacturer’s protocol. Total RNA was then extracted using the RNeasy PowerBiofilm Kit (Qiagen, Hilden, German) and sequenced using Illumina Novaseq platform with 150 bp short-insert library to generate 2 Gb paired-end reads for each sample. The raw reads were trimmed with Trimmomatic v0.36 (Bolger et al., 2014) to remove adapters and low-quality bases with the setting ILLUMINACLIP: TruSeq3-PE.fa:2:30:10 and then mapped to the S. aureus ATCC 43300 genome by using Bowtie2 v2.3.5 (Langmead and Salzberg, 2012).^1^ Salmon v.0.13.1 (Patro et al., 2017) was used to quantify the abundance of successfully mapped transcripts, and differential expression analysis was conducted using Perl scripts align_and_estimate_abundance.pl. and run_DE_analysis.pl. by using the edgeR (Robinson et al., 2010)^73^ method in Trinity v2.8.5 (Grabherr et al., 2011; Haas et al., 2013) toolkits. Transcripts with false discovery rates <0.05 and an absolute fold-change value >2 were defined as differentially expressed genes (DEGs).

### Sample preparation for proteomics analysis

Preformed biofilms were prepared using the same method as those described for transcriptome analysis, treated with 5 μg/ml elasnin (or media for control) for 2, 6 and 12 h, and then rinsed twice. Biofilm matrix and total proteins were extracted as previously described (Sugimoto et al., 2013) with slight modification. In a typical procedure, biofilms were collected from the bottom of the dish, washed with washing buffer consisting of 10 mM Tris–HCl (pH 8.0) and protease inhibitor cocktail (Sigma-Aldrich), and centrifuged at 5,000 × *g* for 10 min. The pellet was dissolved in a matrix-extraction buffer comprising 10 mM Tris–HCl (pH 8.0), 1 M NaCl and protease-inhibitor cocktail followed by incubation at 25°C for 30 min with gentle rotation. The mixture was centrifuged at 5,000 × *g* for 10 min after incubation, and the supernatant was collected as the biofilm-matrix protein. To extract the total protein, we lysed the pellet with B-PER^™^ bacterial protein extraction reagent (Thermo Scientific) according to the manufacturer’s instructions and sonicated it using a Q125 Sonicator (Qsonica) set at 65% amplitude (five blasts each lasting 15 s with 30 s pauses). The supernatant was collected as the total protein after centrifugation. For all proteomics experiments, three biological replicates were performed for each sample, including the control sample.

The Collected proteins were desalted with Thermo Pierce C18 spin tips and digested with trypsin (Pierce^™^ Trypsin Protease, MS Grade) before injecting into the Bruker TimsTOF Pro Massspectrometer (Bruker Headquarters Billerica, MA, United States) with captive spray ion source. Approximately 200 ng of the digested protein was injected into the Bruker nanoElute system and separated on a C18 column (ionoptiks Aurora UPLC column, Part no. AUR2-25075C18A-CSI), and the sample was eluted with a 30 min gradient of 2–95% aqueous acetonitrile containing 0.1% formic acid at a flow rate of 0.3 μl/min. The m/z range recorded in the MS full scan was 100–1,700 Da.

### Sequence database searching and label-free quantification of proteomics data

The data analysis workflow followed a previously described protocol (Sulaiman et al., 2021). The generated raw data were converted to mgf files by Bruker Compass DataAnalysis and subsequently converted to mzML files by msconvert of the ProteoWizard (Kessner et al., 2008). The mzML files were searched using Comet (version 2016.01 rev.2; Eng et al., 2013) with a custom database. In a typical procedure, the genome sequence of MRSA ATCC 43300 was converted into a protein database by using the GeneMark (Lukashin and Borodovsky, 1998) gene prediction tool. Proteins were then annotated using BLASTp from NCBI by using MRSA NCTC 8325 as the protein database. The sequences of common contaminants such as trypsin and human keratins and the decoy sequences generated by shuffling amino acid sequences between tryptic cleavage sites were added to the database. The decoy sequences in the database were used for the false FDR estimation of identified peptides. The search parameters criteria were set as follows: 15 ppm peptide mass tolerance, monoisotopic mass type, fully digested enzyme termini, 0.05 amu fragment bin tolerance, 0 amu fragment bin offset, carbamidomethylated cysteine and oxidated methionine as the fixed and variable modifications. The Search results from Comet were processed using PeptideProphet (Keller et al., 2002), iProphet and ProteinProphet of the Proteomics Pipeline (Deutsch et al., 2010) in the decoy-assisted non-parametric mode. Every mzML run was analysed independently. Protein identifications were filtered at FDR of 0.01 as predicted by ProteinProphet.

Label-free quantification of proteomics data was accomplished by spectral counting by using the parameters in our previous study (Sulaiman and Lam, 2020). Briefly, proteins that were identified in at least two out of three biological replicates were used for label-free quantification by spectral counting. Proteins were quantified using the normalised spectral-abundance factor (NSAF; Paoletti et al., 2006) where the number of peptide-spectrum matches (PSMs) for each protein divided by the length of the corresponding protein was normalized to the total number of PSMs divided by the lengths of protein for all identified proteins. The DEPs were filtered using the following cutoff: average spectral counts of at least three, *p* value for Student’s t-test on the NSAF values of less than 0.05 and fold changes of ±1.5-fold. Moreover, unique proteins that were only detected in the treatment or control samples were retained for analysis, because they are likely to be upregulated or downregulated after elasnin treatment. To minimize false positives, we only focused on uniquely detected proteins with spectral counts greater than 4. Here, we assume that these unique proteins with sufficiently high spectral counts were also induced/upregulated (if detected only in treatment samples and not in control samples) or repressed/downregulated (if detected only in control samples and not in treatment samples).

### Scanning electron microscope analysis of biofilms treated with elasnin

Samples for SEM analysis were prepared as previously described with slight modification (Kong et al., 2018; Boudjemaa et al., 2019). Preformed biofilms on a copper strip surface were treated with elasnin (5 μg/ml) or TSBG for 6 h followed by overnight fixation with 4% (v/v) glutaraldehyde under 4°C. Thereafter, biofilms were dehydrated in a graded ethanol series (30, 50, 70 and 90% v/v with distilled water and thrice with 100% ethanol for 10 min each step), followed by air drying. Samples were then gold-coated using a gold coater Scancoat Six (Edwards, Irvine, CA, United States) and observed using SEM (JSM-6390, JEOL, Akishima, Tokyo, Japan).

### Bioinformatics analysis

PCA was performed to determine the correlation between individuals and the expression level of transcripts on R by using DESeq2 (Love et al., 2014). Functional annotation and enrichment analysis of DEGs/DEPs was performed using The Database for Annotation, Visualization and Integrated Discovery v6.8 (Huang et al., 2009; Sherman and Lempicki, 2009; ease = 0.01). Cluster analysis was constructed to reveal the similarity of gene expression between the control and elasnin-treated groups based on Bray–Curtis distance matrix by using PAST (version 2.0; Hammer et al., 2001). To construct the interaction network between the DEGs/DEPs, we used STRING v11 (Szklarczyk et al., 2019) to predict the protein–protein interactions.

### Gene deletion, transcription inhibition and overexpression of DEGs

The expression of upregulated DEGs was inhibited, and *icaADBC* was deleted using CRISPR/Cas9 system pCasiSA and pCasSA as described previously (Chen et al., 2017). The genes downregulated by elasnin were overexpressed using the tetracycline-inducible expression vector pRMC2 in the relevant *S. aureus* strains. All plasmids, bacterial strains and primers used in this study are listed in Supplementary Tables S2, S3. pRMC2 was obtained from Tim Foster (Corrigan and Foster, 2009; Addgene plasmid #68940; RRID: Addgene 68,940).^2^ pCasiSA was constructed by mutating pCasSA plasmid, and pCasSA was obtained from Quanjiang Ji (Addgene plasmid #98211; RRID: Addgene_98,211).^3^

Constructed plasmid was transported into the wild-type MRSA ATCC43300 by electroporation. Competent cells were prepared as previously described (Chen et al., 2017) and stored at −80°C. For electroporation, 50 μl of competent cells was thawed on ice for 10 min, mixed with 1–2 μg of plasmid and transferred into a 1 mm electroporation cuvette (Bio-Rad, Hercules, CA, United States). Cells were then pulsed at 2.5 kV, 100 Ω and 25 μF and incubated in 1 ml of TSB at 30°C for 1 h, followed by plating on a TSB agar plate containing 7.5 μg/ml chloramphenicol. The plates containing pRMC2 plasmid were incubated at 37°C, whereas plates with pCasiSA, pCasSA and their derivatives were incubated at 30°C. Mutant strains were then used in the relevant MBIC and MBEC assays as described above. All strains containing pCasiSA, pCasSA and their derivatives were incubated at 30°C throughout the entire assay. The PCR confirmation of ica-deleted mutants is shown in Supplementary Figure S2.

### Biochemical-composition study of biofilms

To determine the biochemical composition of the biofilms, we conducted MBIC and MBEC assays as described above with the addition of DNase I (100 U/ml) and proteinase K (100 μg/ml) for eDNA and protein degradation, respectively. We used two *S. aureus* strains, namely, MRSA ATCC 43300 and MSSA ATCC 25923. The ica-deleted mutants were constructed with MRSA and MSSA, and their biofilm formation was tested as described in the MBIC assay without the addition of antibiotics.

### Quantitative real-time PCR

A 3 ml overnight culture of mutant MRSA strains, in which 0.2 μg/ml anhydrotetracycline was added in overexpressed strains, was harvested and stabilised, and the total RNA was extracted as described above. cDNA was then synthesised with RevertAid H Minus First-Strand cDNA Synthesis Kit after removing genomic DNA by using DNase I (Thermo Fisher Scientific Inc., Waltham, MA, United States) followed by quantification on a Roche Diagnostics GmbH LightCycler 480 Instrument II Realtime PCR System using the SYBR Green RT-PCR Reagents Kit (Applied Biosystems). In this process, polymerase activation was carried out at 95°C for 10 min, followed by annealing and extension at 55°C for 1 min for 40 cycles. The specificity of primer pairs for PCR amplification was checked using the melting-curve method. Two biological and three technical replicates were employed for each sample, and the relative gene expression level was calculated based on the 2ΔΔCt using *gyrB* as the internal-reference gene.

### Statistical analyses

Statistical analyses for all data were performed using GraphPad Prism 8.0.2 software and Microsoft Excel 2012 Edition (Microsoft, Redmond, WA, United States).

## Results

### Bioassay-guided isolation of compounds that target biofilm

**Figure 1:**
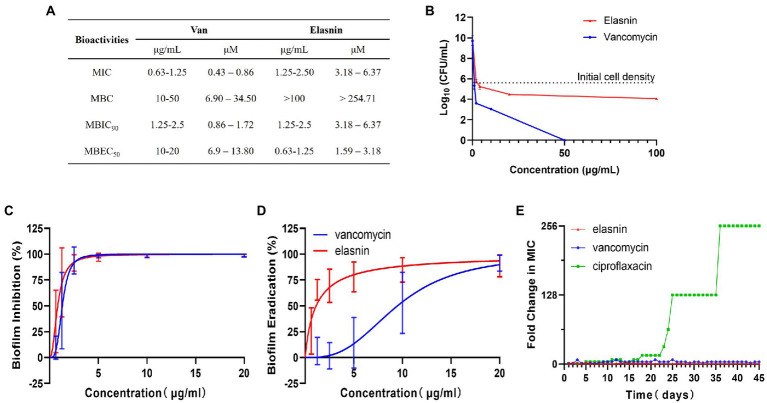
Bioactivities and resistance-development study of elasnin against MRSA. **(A)** Summary of MICs, MBCs, MBICs and MBECs of MRSA towards vancomycin and elasnin. **(B)** Cell viability of MRSA after 24 h of treatment with various concentrations of elasnin and vancomycin (*n* = 3). **(C)** Minimum concentration needed to inhibit 90% of biofilm formation (*n* = 12, average ± standard deviation). **(D)** Minimum concentration needed to eradicate 50% of pre-formed biofilms (*n* = 12, average ± standard deviation). **(E)** Fold change in MICs towards elasnin, vancomycin and ciprofloxacin after 45-days of exposure under sub-inhibitory concentrations (0.5 × MIC) of the respective antimicrobials (*n* = 3).

Secondary metabolites produced by 12 actinobacterial strains under different culture conditions were assessed for bioactivities against Gram-positive bacteria (MRSA) and Gram-negative bacteria (*E. coli*, E. coli). This was followed by bioassay-guided fractionation which led to the isolation of three antimicrobial compounds (e.g., xanthone, hitachimycin, and resistomycin) and the antibiofilm compound - elasnin, which was isolated from Streptomyces mobaraensis DSM 40847 and showed potent activity against MRSA (Supplementary Table S1). To assess elasnin’s activity, we compared purified elasnin with vancomycin in terms of MIC, MBC, MBIC, and MBEC against MRSA. The MIC values reflect the antibiotics’ antimicrobial activities against planktonic cells. Results show that MRSA was susceptible to vancomycin (MIC of 0.63–1.25 μg/ml) and elasnin (MIC of 1.25–2.5 μg/ml, Figure 1A). The MBC values measure the compounds’ killing effect on cells. Vancomycin exhibited strong bactericidal activities in a concentration-dependent manner and had MBC values ranging from 10 to 50 μg/ml, whereas elasnin showed bacteriostatic activity, had a higher MBC value than vancomycin, and did not cause significant changes in cell density in the concentration of more than 100 μg/ml (Figures 1A,B). The MBIC values represent the ability of the compounds to inhibit biofilm formation, whereas the MBEC values indicate the ability to eradicate pre-formed mature biofilms. Elasnin and vancomycin showed strong biofilm-inhibiting activities against MRSA with MBIC_90_ values of 1.25–2.5 μg/ml (Figure 1C). The pre-formed biofilms showed strong resistance to vancomycin with MBEC_50_ of 10–20 μg/ml. However, they can still be eradicated with elasnin at a much lower MBEC_50_ between 0.63–1.25 μg/ml (Figure 1D). When being tested in a LCWB model that mimics chronic wound biofilms, both elasnin and vancomycin showed an increase in the effective concentrations with MBIC_90_ of 250–500 and 20–100 μg/ml, respectively, and with the same MBEC_50_ of 100–500 μg/ml (Supplementary Figure S3). Overall, elasnin exhibited higher effectiveness in biofilm inhibition and especially in biofilm eradication, relative to its activities against planktonic cells. Cells can still proliferate after being exposed to elasnin, suggesting that elasnin could be used as a biofilm-targeting compound that interferes with biofilm formation and maintenance rather than killing the planktonic cells.

In addition, in the test for the potential development of resistance, the MIC of MRSA treated with elasnin did not change over a period of 45-days (Figure 1E; Supplementary Table S4), suggesting that the cell susceptibility to elasnin treatment did not change. Elasnin also did not show any cytotoxicity against Neuro2 cell lines at a concentration of 10 μg/ml (Supplementary Figure S4A) or HT22 cells at concentrations of up to 25 μg/ml, which is 10 times its MBIC_90_ and MBEC_50_ (Supplementary Figure S4A). Elasnin’s effect on cell viability was only observed at concentrations higher than 25 μg/ml (Supplementary Figure S4B).

### Elasnin destroyed the biofilm matrix

**Figure 2:**
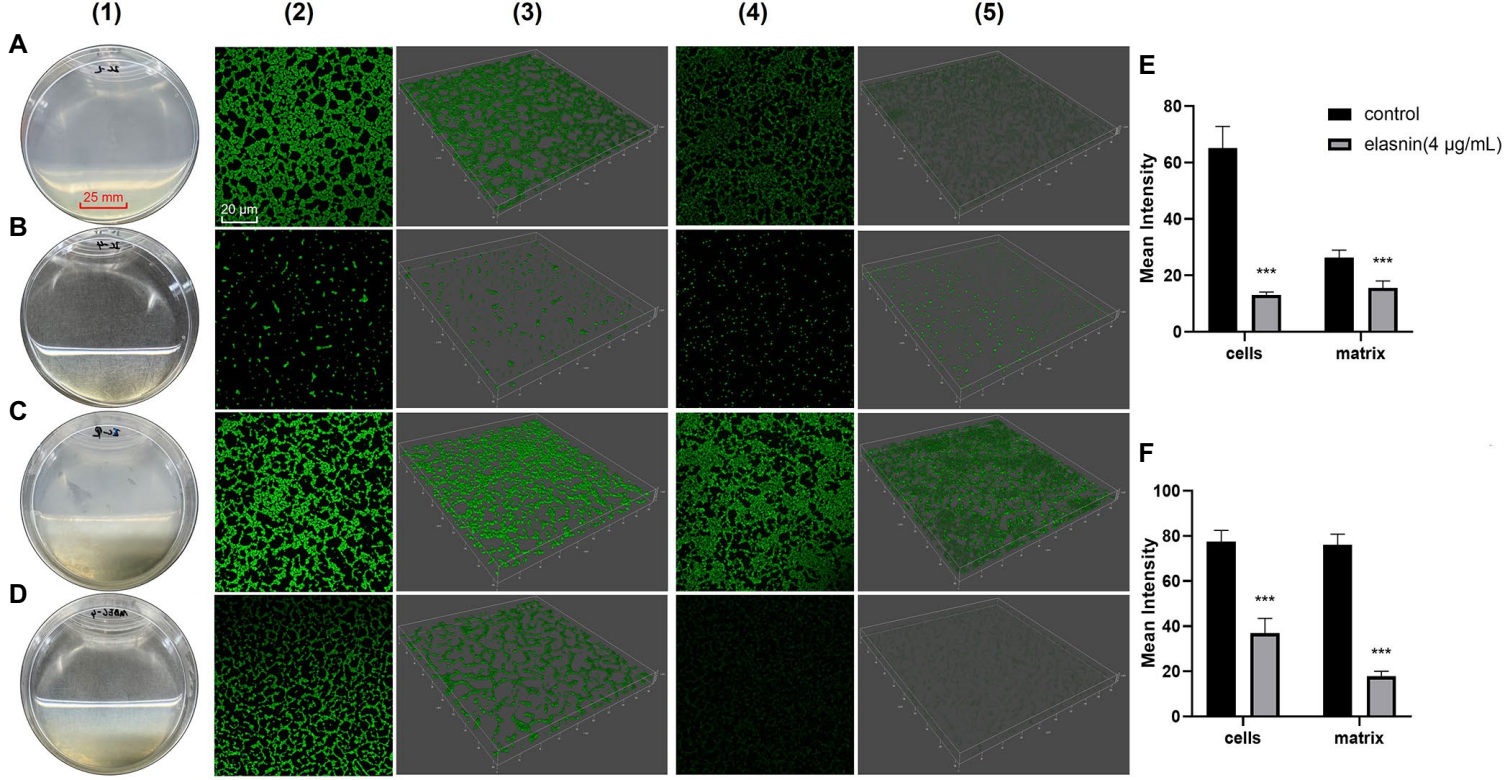
Comparison between the effect of elasnin on MRSA biofilm cells and matrix. **(A)** Image of the biofilms after incubation for 24 h (control). **(B)** Image of the biofilms after incubation for 24 h with 4 μg/ml elasnin. **(C)** Image of the pre-formed biofilms after another incubation for 18 h (control). **(D)** Image of the pre-formed biofilms after another incubation for 18 h with 4 μg/ml elasnin. **(E)** Quantitative analysis of confocal images acquired in biofilm-inhibition assay and **(F)** biofilm-eradication assay. Differences between different groups were calculated by Student’s *t*-test and are indicated by asterisks as follows: ^***^for *p* < 0.001. Series 1 show the images of biofilms under direct observation. Series 2 and 3 are the two-and three-dimensional confocal images of biofilm cells stained with FilmTracer^™^ FM^®^ 1–43 green biofilm cell stain, respectively. Series 4 and 5 are the 2D and 3D images of biofilm matrix stained by FilmTracer^™^ SYPRO^®^ Ruby Biofilm Matrix Stain, respectively. Confocal images were acquired under the same conditions, and quantitative analysis was conducted using Leica Application Suite X based on the relative fluorescence of 3D confocal images.

CLSM was used to observe the effect of elasnin on biofilm structures. Biofilm-inhibition assay showed that untreated biofilms had distinct shapes with a high density of organised cells and matrix (Figure 2A), whereas the elasnin-treated biofilms exhibited a significant decrease in cell density and matrix and both biofilms were randomly distributed (Figure 2B). Biofilm-eradication assay revealed that the pre-formed biofilms were eradicated by elasnin because most of the biofilm cells were released into the medium (Figure 2D). CLSM images demonstrated that the distribution patterns of the cells changed after elasnin treatment, in which the untreated biofilm cells were distributed as clumps with rough edges (Figure 2C), whereas elasnin-treated biofilm cells were distributed as narrow strips with smooth edges (Figure 2D). Similarly, the high density of organised biofilm matrix became sparse and scattered after elasnin treatment. Quantitative analysis showed that the biofilm cells and matrix were significantly reduced after treatment. In comparison with untreated biofilms, elasnin-treated ones exhibited ~80 and 35% decrease in cell density and matrix in the biofilm-inhibition assay (Figure 2E). In the biofilm-eradication assay, cells and matrix densities decreased by over 50 and 70% (Figure 2F).

### Gene expression of virulence factors and products in the extracellular region were downregulated following elasnin treatment

**Figure 3:**
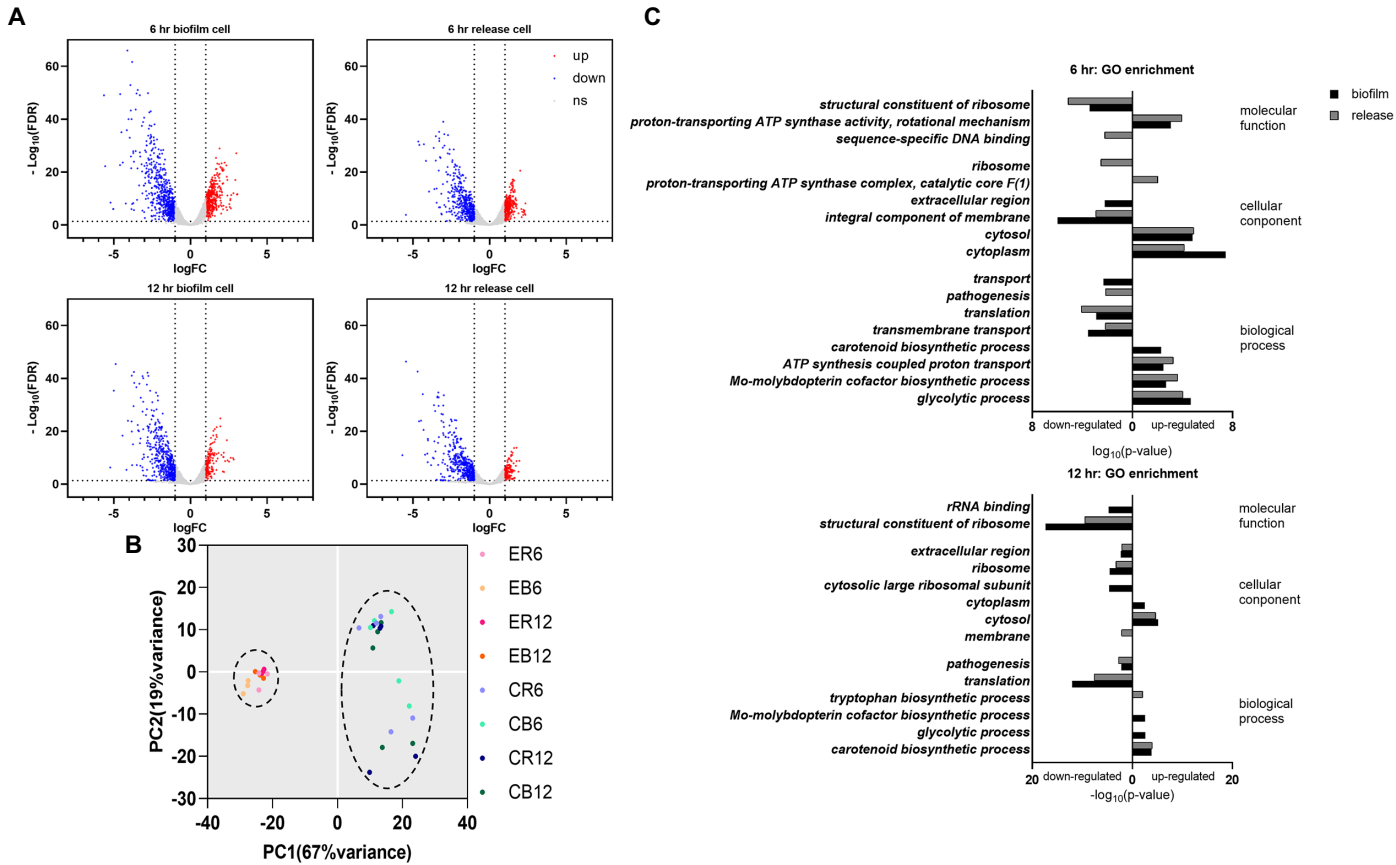
Changes in the gene expression of MRSA cells after elasnin treatment. **(A)** Volcano plot of RNA-seq profiles of MRSA cells showing the altered gene expression pattern of collected samples (up: upregulated, corresponding to the red dots; down: downregulated, corresponding to the blue dots; ns: not significantly changed, corresponding to the gray dots). **(B)** Principal component analysis of RNA-seq samples (treated with elasnin and untreated), in which elasnin treatment mainly caused the altered gene expression. ER: cells released from elasnin-treated biofilms; CR: cells naturally released from the biofilms; EB: elasnin-treated biofilms cells; CB: biofilm cells. The numbers following the letters indicate the duration of elasnin treatment (in hour). **(C)** Gene ontology enrichment analysis of differentially expressed genes (in biofilm and released cells, marked by black and gray bars, respectively), highlighting the alterations in genes for pathogenesis and those located in the extracellular region and the integral component of the membrane.

Among the 2,791 detected gene transcripts, 1,010 were differentially expressed (≥2.0-fold change in gene expression) on MRSA biofilm cells treated with elasnin for 6 h compared with untreated biofilm cells (control). The percentage of eradicated cells after 6 h of treatment is shown in Supplementary Figure S1B. The number of differentially expressed genes (DEGs) decreased to 668 when the treatment time was extended to 12 h. For cells released from the biofilms, 720 and 609 genes were differentially expressed between the treatment and control groups at 6 and 12 h, respectively (Figure 3A). Principal component analysis showed a clear separation between the clusters of elasnin-treated and untreated samples along the PC1 axis, indicating that elasnin treatment mainly accounts for the differences in gene expression (Figure 3B).

DEGs were then processed using gene ontology (GO) enrichment analysis in terms of their molecular function, cellular component, and biological process (Figure 3C). In the samples treated for 6 h, amongst the enriched GO terms of downregulated genes, the cellular component of the extracellular region was observed only for biofilm samples, whereas the biological process of pathogenesis was shown only in released cells samples. Additionally, the GO terms of translation, transmembrane transport and integral component of the membrane were downregulated in biofilm and released cells. In the 12 h treated samples, downregulated genes were enriched in the extracellular region and pathogenesis in the biofilm and released cells, whereas membrane components were observed only in the released cells.

**Figure 4:**
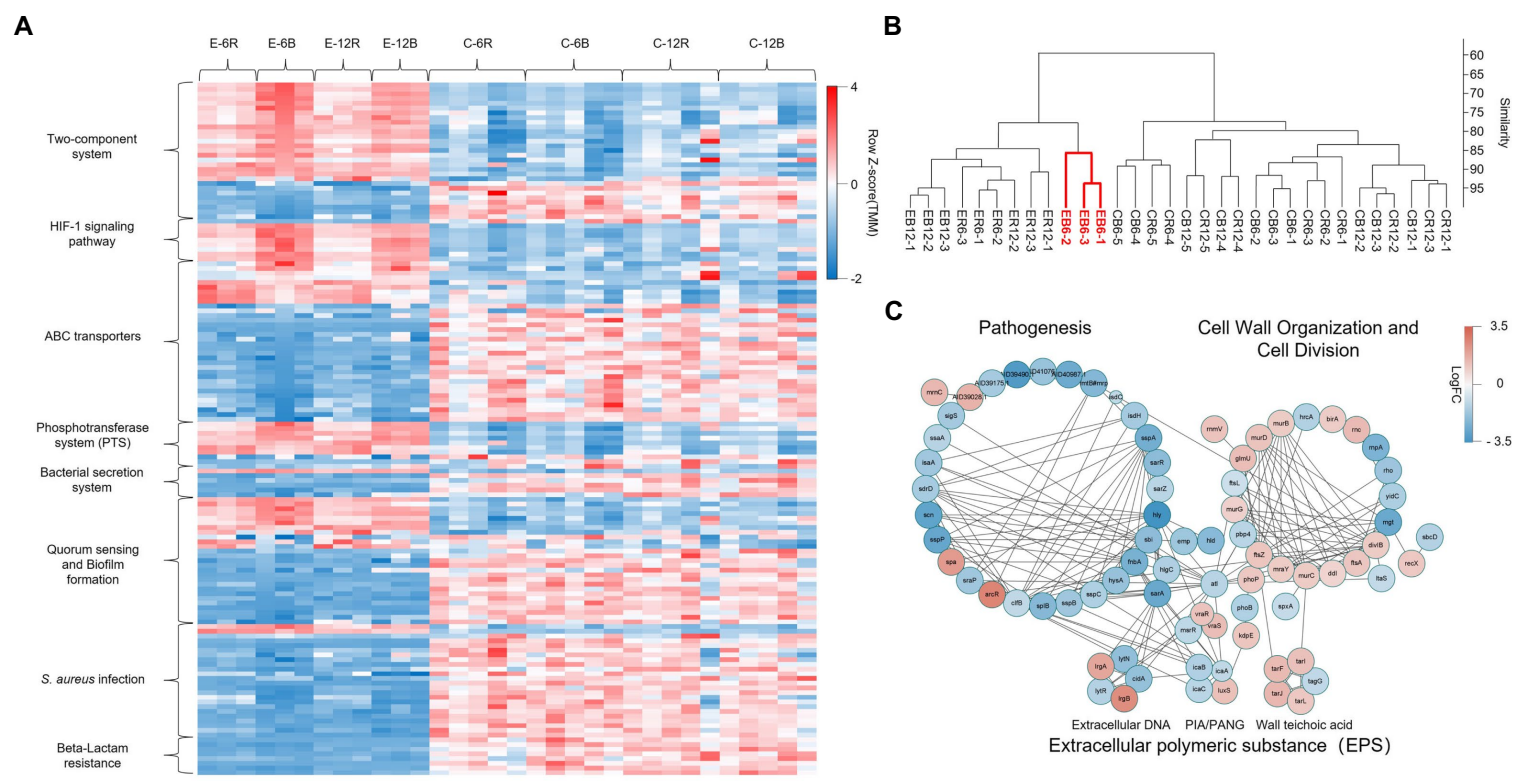
Gene expression of the MRSA biofilm cells treated for 6 h having the most distinct one among all groups. **(A)** Heatmap of the expression level of DEGs for selected KEGG pathways, revealing the increased signal transduction, reduced membrane transport, quorum sensing, *Staphylococcus* infection and β-lactam resistance in elasnin-treated samples. **(B)** Hierarchical clustering of the RNA-seq data by using the normalised reads count, revealing that biofilm cells exhibited the most unique gene expression pattern after 6 h of elasnin treatment. **(C)** Networks of DEGs of biofilm cells after 6 h of elasnin treatment and their functional associations. The nodes represent the differentially expressed genes between the control and treatment groups after 6 h of treatment, and the edges indicate their associations as predicted by STRING. Red colour indicates upregulated gene expression, whereas blue colour indicates downregulated gene expression following elasnin treatment. The size is inversely proportional to the *p* value, as described in the Material and Method section (a larger node corresponds to a smaller *p* value).

Figure 4A shows the gene expression in selected pathways of DEGs that were analysed using the Kyoto Encyclopaedia of Genes and Genomes (KEGG) pathway assessment. Signal transduction-related genes (two-component system and HIF-1 signalling pathway) were overexpressed in the elasnin treatment group, whereas many genes related to membrane transport (ABC transporters, phosphotransferase system, and bacterial secretion system), quorum sensing, and especially Staphylococcus infection and β-lactam resistance were downregulated. The results of hierarchical clustering of gene expression data (Figure 4B) revealed that biofilm cells exhibited the most unique gene-expression pattern after 6 h of elasnin treatment. Subsequently, the biofilm samples treated for 6 h were used to build the gene interaction network (Figure 4C) which shows that elasnin-treated biofilm cells exhibited low expression levels of genes involved in pathogenesis, including global regulon (*sarA* and RNAIII), EPS production (*icaA, icaB, and icaC*), murein hydrolases and autolysins (*atl, lytR, and cidA*), serine protease (*sspA, sspB, sspC, sspP, and splB*), toxin (*hld, hlgC, and hly*), and adhesins (*fnbA, clfB, sdrD, and emp*). Interestingly, genes related to cell wall organisation and cell division, that is, *murB, murC, murD, mraY, diviB, and ftsZ*, were upregulated after 6 h of elasnin treatment.

### Effects of elasnin on the cell cycle and EPS production of MRSA and cell resistance to penicillin G

**Figure 5:**
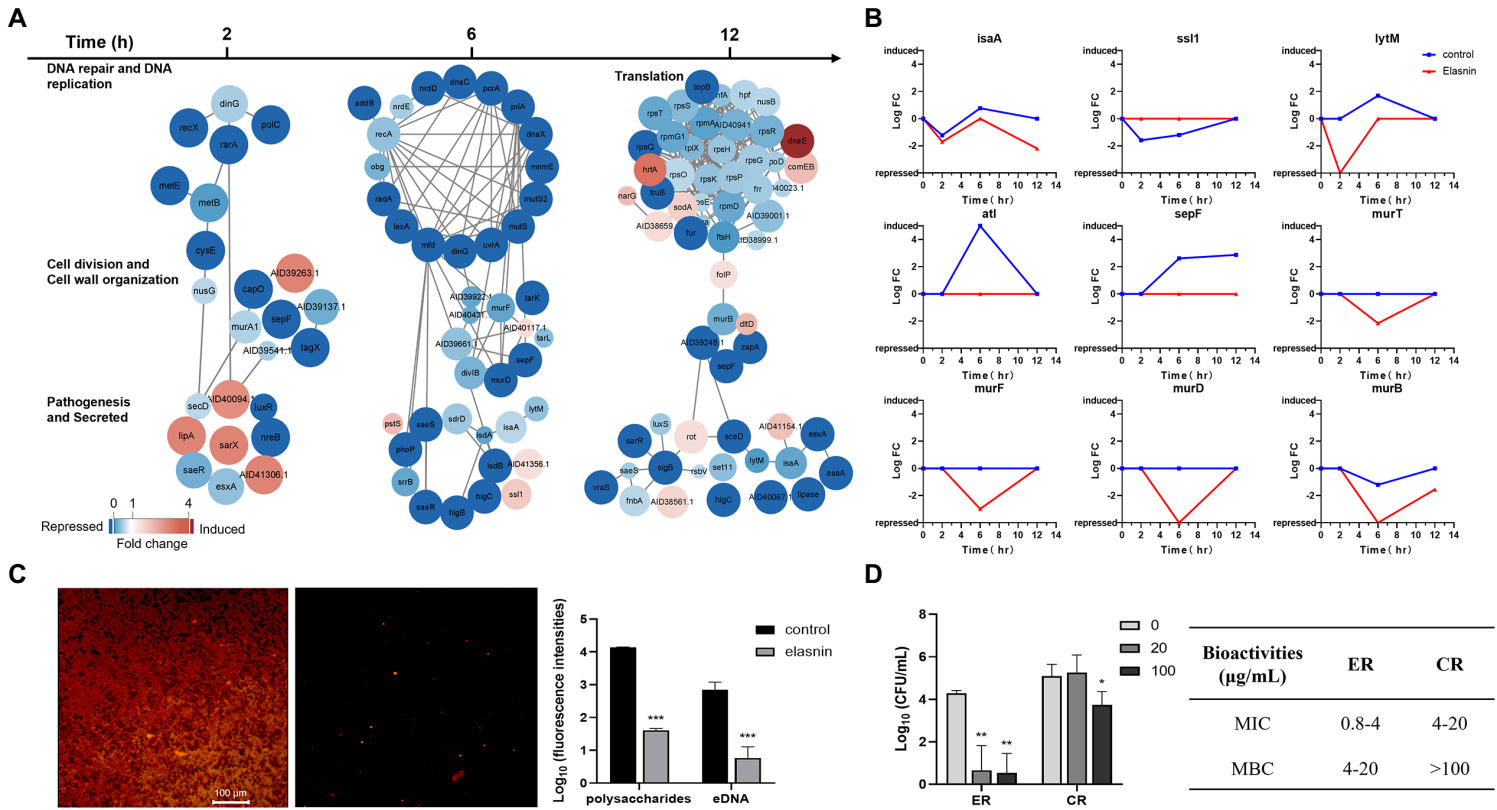
Elasnin interfered with cell cycle and EPS production and reduced the antibiotic resistance of MRSA. **(A)** Differentially expressed proteins in selected functional subsets after 2, 6 and 12 h of elasnin treatment. **(B)** Changes in the expression level of selected DEPs during the biofilm eradication with elasnin. **(C)** Effect of elasnin on the production of polysaccharides (red, stained with Concanavalin A) and eDNA (green, stained with TOTO-1) of MRSA biofilm, as visualised by confocal microscopy. Fluorescence intensities were calculated based on acquired 3D confocal images (*n* = 3). Here, the polysaccharides and the eDNA were stained simultaneously. A separate staining image of polysaccharides alone and eDNA alone is shown in Supplementary Figure S4D. **(D)** Antibiotic susceptibility of MRSA cells against Penicillin G after being released from the biofilms (*n* = 3). ER are the cells released from the biofilms after elasnin treatment, and CR are the cells released naturally (without any treatment). Differences between different groups were calculated by Student’s *t*-test and are indicated by asterisks as follows: ^*^for *p* < 0.05, ^**^for *p* < 0.01 and ^***^for *p* < 0.001.

To further investigate the mode of action of elasnin in the growth inhibition and biofilm eradication of MRSA, we used label-free quantitative proteomics analysis to study the protein expression dynamics of MRSA biofilm cells during biofilm eradication after elasnin treatment (Figure 5A). The analysis revealed that 105 proteins were differentially expressed in the MRSA biofilm cells treated with elasnin for 2 h compared with untreated samples. The proteins involved in DNA repair and replication, cell division and cell wall organisation, pathogenesis (e.g., virulence regulator *SarX* and SaeR), and secreted virulence factors (EsxA) were down-regulated and repressed. Alternately, amidohydrolase (AID39263.1) and secreted lipase, and dipeptidyl-peptidase (AID41306.1) were upregulated. After 6 h of treatment, the number of DEPs reached 250, which was the highest among all time points (2 and 12 h). At 6 h, elasnin downregulated or repressed numerous proteins involved in DNA repair and replication, cell division, cell wall organisation, and the production of virulence factors (e.g., adhesin SdrD, toxin HlgB and HlgC and autolysin LytM). On the other hand, the expression levels of secreted peptidase (Staphylococcal superantigen-like 1 [Ssl1]) and amidase (AID41356.1) were upregulated. When the treatment duration was prolonged to 12 h, the number of DEPs decreased to 154, and most DEPs were primarily related to translation and pathogenesis, with a few involved in cell division and cell wall organisation. Except for LytM and lipase, no other hydrolases, lyases, and proteins involved in DNA replication and repair were differentially expressed.

Changes in the expression levels of several hydrolases and proteins related to cell division and cell-wall organisation in the control samples showed the abundance of hydrolases encoded by IsaA, LytM, and Atl reached the highest at 6 h, and the abundance of Ssl1 should be reduced during this period (Figure 5B). However, elasnin treatment reduced the abundance of IsaA and LytM, repressed the expression of autolysin (Atl), and stopped the changes in Ssl1. In the control samples, the abundance of SepF exhibited a continued increase from 2 h to 12 h, and the abundance of proteins involved in cell wall biogenesis (Mur family proteins) remained stable. However, elasnin-treated biofilm cells exhibited decreased abundance in Mur family proteins after 6 h of treatment, and SepF was repressed throughout the entire process.

The effect of elasnin treatment on biofilm matrix composition was shown in Figure 5C and Supplementary Figure S5. The number of polysaccharides and eDNA in the biofilm matrix was remarkably reduced after elasnin treatment. Cells released from the biofilms were collected for MIC and MBC assay by using a β-lactam antibiotic, penicillin G. Consistent with the transcriptomic analysis results, the released cells treated by elasnin (ER, Figure 5D) exhibited lower resistance to penicillin G in which the MIC (0.8–4 μg/ml) and MBC (4–20 μg/ml) decreased by approximately five times compared to the MIC (4–20 μg/ml) and MBC (>100 μg/ml) of naturally released cells (CR; Figure 5D).

### Effects of elasnin on the cell wall of MRSA cells, and role of *sarZ* on elasnin-induced biofilm eradication

**Figure 6:**
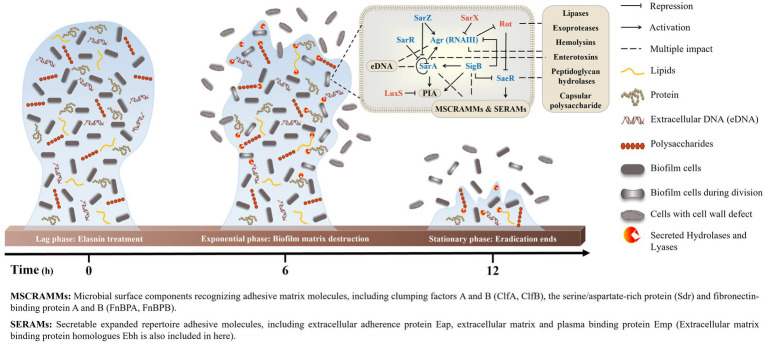
Proposed model of MRSA-biofilm eradication by elasnin. The peak of eradication occurred in the exponential phase, when a large amount of biofilm cells was undergoing cell division. Elasnin inhibited the expression of virulence regulons that controls virulence factors, such as adhesive proteins, cell-wall hydrolases and EPS, thereby interfering with cell division and generating cell-wall-defective cells. Moreover, the increased production of hydrolases and lyases degraded the old EPSs and subsequently released the biofilm cells from the biofilms.

**Figure 7:**
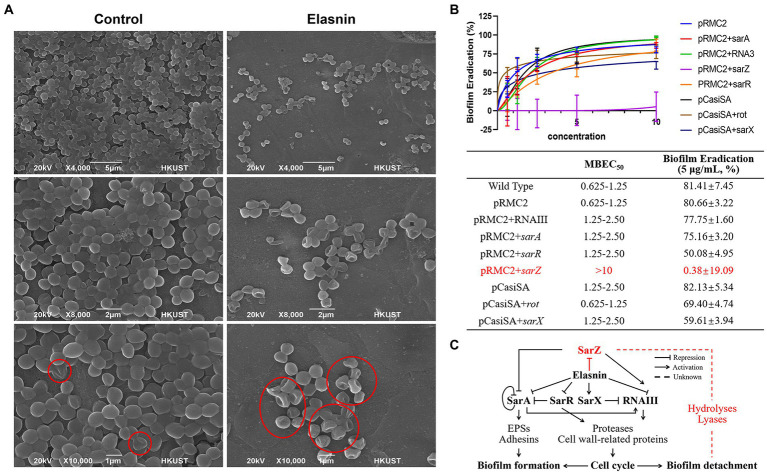
Validation of the proposed mode of action of elasnin. **(A)** Scanning electron microscopy observation of untreated and elasnin-treated (5 μg/ml) biofilm cells, illustrating the large amount of defective cells within elasnin-treated biofilms. Regions marked with red outlines indicate cells with cell wall defect. **(B)** MBEC and biofilm-eradication efficacy of elasnin (5 μg/ml) against transcription-inhibited and overexpressed strains (*n* = 9, average ± standard deviation). The expression of overexpressed genes was induced by 0.2 μg/ml anhydrotetracycline. **(C)** Schematic of the regulatory network affected by elasnin during MRSA biofilm formation and biofilm detachment. This figure highlights the important role of *SarZ* in regulating degradative enzymes during elasnin-induced biofilm eradication.

Transcriptomics and proteomics analysis revealed that combined with the increased sensitivity of elasnin-treated cells towards penicillin G, elasnin may also interfere with the proper cell division and cell wall organisation process during the exponential phase when numerous cells are undergoing cell division (Figure 6). To validate our hypothesis that elasnin causes cell wall defects in biofilm cells, the morphological changes in MRSA cells after 6 h of exposure were examined under SEM (Figure 7A). Consistent with our hypothesis, untreated biofilms showed a dense layer of normal grape-like cell clusters, whereas elasnin-treated biofilms were scattered, in which a majority of cells exhibited a defective appearance with clear collapses around the centre of the cell. Interestingly, defective cells were observed in the untreated biofilms, but they accounted only for less than 1% (2 of ~200) of the total cells, whereas the proportion of defective cells was more than 70% (25 of 34) in elasnin-treated biofilm.

Considering the complex regulatory network of virulence factors, we determined the main determinant by comparing the biofilm-eradication activity of transcription-inhibited mutants and overexpressed strains (Figure 7B). The regulators (e.g., *rot and sarX*) induced following elasnin treatment were transcriptionally inhibited by CRISPR/Cas9 transcription inhibition system pCasiSA, whereas the downregulated genes (e.g., *sarA, sarZ, sarR*, and RNAIII) were overexpressed with the expression vector pRMC2 (see RT-qPCR results in Supplementary Figure S6). In comparison with the control strains (strains with empty plasmids), the mutants of *sarA, sarR*, and RNAIII showed an increased MBECs of 1.25–2.5 μg/ml, and a decreased eradication rates varied from 50.1 to 77.8%. MBEC_50_ did not increase in mutants of *rot* and *sarX*, but the eradication efficiency (5 μg/ml) of elasnin was reduced to 69.4 and 59.6%, respectively. Among all the mutants, the *sarZ*-overexpressed mutant showed the highest resistance to elasnin with an MBEC of above 10 μg/ml and an eradication rate of almost 0%, and the ability of elasnin to inhibit the biofilm formation of the *sarZ*-overexpressed mutant was largely reduced at an MBIC_90_ above 10 μg/ml (Supplementary Table S5). Simultaneously, the *sarZ*-deleted MRSA mutant NE567 exhibited increased sensitivity to elasnin treatment with reduced values in MBIC_90_ and MBEC_50_, respectively, (Supplementary Figure S7). All other mutants showed resistance to elasnin in the biofilm-formation inhibition assay with MBIC_90_ of 5–10 μg/ml for the mutants of *sarA, rot*, and *sarX*, and MBIC_90_ higher than 10 μg/ml for the mutants of *sarR* and RNAIII. Interestingly, the overexpressed mutants of *sarR*, RNAIII, and especially *sarZ* showed a remarkable reduction in terms of biofilm formation compared to the control strains and the wild-type strain. To conclude, the effect of elasnin on virulence regulons affected biofilm formation, and the repression of *sarZ* was responsible for the biofilm eradication (Figure 7C).

## Discussion

The selective pressure exerted by antimicrobials enriches the naturally existing antibiotic-resistant bacteria in the environment, thereby accelerating the development of resistance (Martinez, 2009; Holmes et al., 2016). Considering the protection provided by the biofilm matrix and the intense gene transfer and differentiation within the biofilm, compounds that simply kill cells but leave the biofilm matrix intact for microbial utilisation are likely to boost resistance development. With the inevitable increase in antibiotic resistance and the considerable challenges in biofilm-associated antimicrobial therapy, effective antibiofilm agents, particularly those that can effectively eradicate established biofilms, are urgently needed. In the present work, we conducted bioassay-guided compound isolation to identify the biofilm-targeting compounds that can effectively inhibit and eradicate the biofilms without killing the cells. Elasnin was identified as a potent biofilm-eradicating drug candidate against MRSA. It eradicates biofilms by destroying the biofilm matrix and does not remarkably affect the viability of the released cells. Therefore, theoretically, the risk of cells developing reduced susceptibility towards elasnin is low, as confirmed by the non-observable increase in MIC upon subjecting MRSA cells to continuous serial passaging in the presence of sub-inhibitory concentrations of elasnin. The cells released following elasnin treatment were also more susceptible to the β-lactam antibiotic penicillin G. Elasnin further exhibited low cytotoxicity towards different cell lines, which is consistent with a previous study (Ohno et al., 1978). Elasnin was effective in a chronic wound biofilm model with the presence of blood and plasma, indicating the therapeutic potential of elasnin as a safe and potent biofilm-eradicating agent against MRSA biofilm. Therefore, elasnin harbors great potential to be developed as a disinfectant for biofilm removal and to be used for treating chronic wounds.

The mechanism in which elasnin eradicates the established MRSA biofilm was then elucidated through a series of analyses (Figure 6). As a part of pathogenesis, the production of adhesive proteins and PIA or poly-β(1–6)-N-acetylglucosamine encoded by ica operon was essential for the biofilm formation of MRSA ATCC 43300 (Supplementary Figure S8B). Moreover, the maintenance of the established biofilms required the participation of adhesive proteins and eDNA (Supplementary Figures S8A,C) and the cell cycle and cell wall hydrolases (LytM and autolysin) that are essential for cell separation, which are all regulated by the virulence regulons. Therefore, many proteases and cell wall hydrolases are expressed during the exponential phase when biofilms undergo proliferation and maturation. However, since elasnin represses the virulence regulons, the production of some cell wall hydrolases, adhesins and EPSs was inhibited during treatment. Consequently, cells could not properly divide, and thus, very limited EPSs were synthesised. Meanwhile, some lipase and proteases were largely secreted, causing the degradation of the existing EPSs and the destruction of the biofilm matrix. The biofilm cells were released back to the media, and most of them had defective cell wall structures caused by the inhibition of the cell division process and the down-regulation of the expression of cell wall-related proteins. Consequently, cells released from the elasnin-treated biofilms showed increased susceptibility to the β-lactam antibiotic penicillin G, highlighting the possible application of elasnin in combinatorial treatment for rescuing old drugs that had become ineffective due to resistance.

*Staphylococcus aureu* has a very complex regulatory network of biofilm formation and virulence expression, in which many important regulators are affected by elasnin. Accordingly, multiple mutants were generated to reveal the key determinants by comparing elasnin’s activities against the mutants. All mutants showed increased ability in resisting elasnin in the biofilm inhibition assay. The overexpression of *sarA* can increase the production of EPSs and adhesins assisting with biofilm maturation, whereas the overexpression of *sarR* and RNAIII and the repression of *sarX* can increase the production of proteases and cell wall-related proteins for cell division. *sarZ* activates the expression of RNAIII, represses *sarA*, and regulates the production of proteases independently (Ballal et al., 2009; Tamber and Cheung, 2009). The overexpression of *sarZ* restored the cell’s ability to divide to some extent (Supplementary Table S5; Figure S7E) possibly by repressing *sarA* and increasing the production of proteases and cell wall-related proteins through or independent from *sarA* and RNAIII (Figure 7C). Consequently, these mutants exhibited increased resistance to the biofilm inhibition caused by elasnin. Some of these mutants (e.g., RNAIII, *sarR*, and *sarZ* overexpressed mutant) showed reduced ability to form biofilms, perhaps due to the increased production of proteases and hydrolases (Figure 7C; Supplementary Table S5).

Elasnin lost its efficacy only in eradicating the established biofilm of mutants overexpressed with *sarZ* and exhibited increased activity against the *sarZ*-deleted mutant (Supplementary Figures S7A,B), suggesting the crucial role of *sarZ* during the elasnin-induced biofilm eradication. Based on our results, deletion of *sarZ* does not influence elasnin’s efficiency in cell growth inhibition (Supplementary Figures S7D,E), suggesting increased antibiofilm activity is not related to elasnin’s inhibition of cell division. Moreover, we found that MRSA had higher dependence on adhesive proteins for biofilm formation and maintenance than MSSA (Supplementary Figure S8C; Table S6), and elasnin exhibited higher antibiofilm activities against MRSA than MSSA (Supplementary Table S7). Therefore, elasnin-induced degradation of existing adhesive proteins is crucial for eradicating the established biofilms, and stopping biofilm eradication (as shown in the *sarZ*-overexpressed mutant; Figure 7B) required the inhibition of the matrix destruction caused by the increase in degrading exoproteins. However, the role of *sarZ* in this process has not been reported. Therefore, we proposed that (i) *sarZ* may be a repressor of the production of the degrading exoproteins, or (ii) the proteases upregulated by *sarZ* inactivate the corresponding exoproteins, and this regulatory pathway was independent of the above-mentioned regulons.

Biofilm development and its regulation has been extensively studied, but gaping holes remain to be filled. Previous studies have reported that increased SarA level and/or reduced protease production in a *sarZ*-deleted MRSA mutant resulted in increased biofilm formation (Tamber and Cheung, 2009), which is consistent with our observation of reduced biofilm formation in the *sarZ*-overexpressed mutant. Meanwhile, the increased expression of degradative enzymes like lipases and putative hemolysin reported in the *sarZ*-deleted Staphylococcus epidermidis (Wang et al., 2008) was also observed in the elasnin-treated biofilm cells when *sarZ* was repressed, which is believed to be responsible for the elasnin-induced biofilm eradication. Staphylococci produce different factors for different stages of infection (colonization, invasion, proliferation, dissemination, etc.), and biofilm development assist in these different stages (biofilm formation assist in colonization, whereas biofilm dispersion/detachment assist in dissemination). Considering the regulatory effect of *sarZ* on biofilm development in *S. aureus* and *S. epidermidis*, *sarZ* might be an important regulator that controls the transition among different stages, especially that *sarZ* exerts a regulatory effect on several traits and is expressed differentially at different growth phases (Kaito et al., 2006; Ballal et al., 2009; Chen et al., 2009). Yet, the exact regulations on how *sarZ* governs biofilm development are still unclear, and therefore, detailed studies on the regulations of *sarZ* during staphylococcal biofilm development should be further investigated. Besides, the roles of elasnin in intra-and intercell communications are also worth exploring, because elasnin is produced by multiple Streptomyces species and has a similar chemical structure to photopyrones, the novel quorum-sensing signals in Photorhabdus.

To conclude, the present study discovered elasnin as a potent biofilm-eradicating compound and elucidated its mode of action. Elasnin destroyed the biofilm matrix of MRSA and reduced cells’ resistance to penicillin G, exhibiting low cytotoxicity and a low risk of resistance development. Mechanistic study revealed that elasnin repressed the expression of virulence factors and increased the secretion of degradative exoproteins, thus inhibiting cell division and leading to the degradation of the biofilm matrix. Through genetic manipulation assay, we determined the role of affected regulons during the elasnin-induced biofilm eradication and discovered that *sarZ* is an attractive target for Staphylococcal biofilm eradication. Overall, our study identified elasnin as a potent anti-virulence and biofilm-eradicating compound that harbors great potential in controlling MRSA biofilms with reduced risks in resistance development and provided new insights into the molecular targets for the discovery of MRSA biofilm eradicator.

## Data availability statement

The datasets presented in this study can be found in online repositories. The names of the repository/repositories and accession number(s) can be found at: https://www.ncbi.nlm.nih.gov/genbank/, PRJNA740277 and http://www.proteomexchange.org/, PXD026836.

## Author contributions

LL designed and carried out the experiments, did transcriptomic and related bioinformatics analysis, interpreted the data, and prepared the manuscript. JS did the proteomic analysis, wrote related methods, and did revision of the manuscript. YX did the PCA and bioinformatics analysis and wrote related methods. AC did the cytotoxicity test, interpreted the data, and wrote related methods. RW did the cluster analysis and wrote related methods. JM, WW, and WL helped in the experiments and data interpretation. Y-XL helped in experiment design. FC gave technical support. HL and P-YQ supervised this study, gave technical support and conceptual advice, and did the major edition of the manuscript. All authors reviewed and edited the manuscript, contributed to the article, and approved the submitted version.

## Funding

This work was financially supported by the National Key R&D Program of China (2018YFA0903200), the Key Special Project for Introduced Talents Team of Southern Marine Science and Engineering Guangdong Laboratory (Guangzhou, GML2019ZD0409), the China Ocean Mineral Resources Research and Development Association (COMRRDA17SC01), the Hong Kong Branch of Southern Marine Science and Engineering Guangdong Laboratory (Guangzhou, SMSEGL20SC01), a CRF grant from the HKSAR government (C6026-19G-A), and the Research Grant Council of the Hong Kong Special Administrative Region, and China (grant no. 16102821).

## Conflict of interest

The authors declare that the research was conducted in the absence of any commercial or financial relationships that could be construed as a potential conflict of interest.

## Publisher’s note

All claims expressed in this article are solely those of the authors and do not necessarily represent those of their affiliated organizations, or those of the publisher, the editors and the reviewers. Any product that may be evaluated in this article, or claim that may be made by its manufacturer, is not guaranteed or endorsed by the publisher.

## Footnotes

^1^ https://genomes.atcc.org/genomes/79691302ed634fef?_ga=2.259377226.1584810311.1616483300-1172888945.1616483300

^2^ http://n2t.net/addgene:68940

^3^ http://n2t.net/addgene:98211

## Abbreviations

MRSA: Methicillin-resistant *Staphylococcus aureus*
MSSA: Methicillin-sensitive *S. aureus*
EPS: Extracellular polymeric substances
eDNA: Extracellular DNA
MIC: Minimum inhibitory concentration
MBC: minimum bactericidal concentration
MBIC: Minimum biofilm inhibitory concentration
MBEC: Minimal biofilm eradication concentration
LCMB model: Lubbock chronic wound biofilm model
TSBG: TSB complemented with 0.5% glucose
DEGs/DEPs: Differentially expressed genes/proteins
CLSM: Confocal laser scanning microscopy
SEM: Scanning electron microscope
GO: Gene ontology
KEGG: Kyoto Encyclopaedia of Genes and Genomes
