# Supplementary material for: Mode of action of elasnin as biofilm formation eradicator of methicillin-resistant Staphylococcus aureus
Source: Front Microbiol. 2022 Aug 8;13:967845. doi: 10.3389/fmicb.2022.967845 (PMC9393526; doi:10.3389/fmicb.2022.967845)
Supplement: Supplementary file 1 [file Data_Sheet_1.docx]

Supplementary Material

## Supplementary Figures


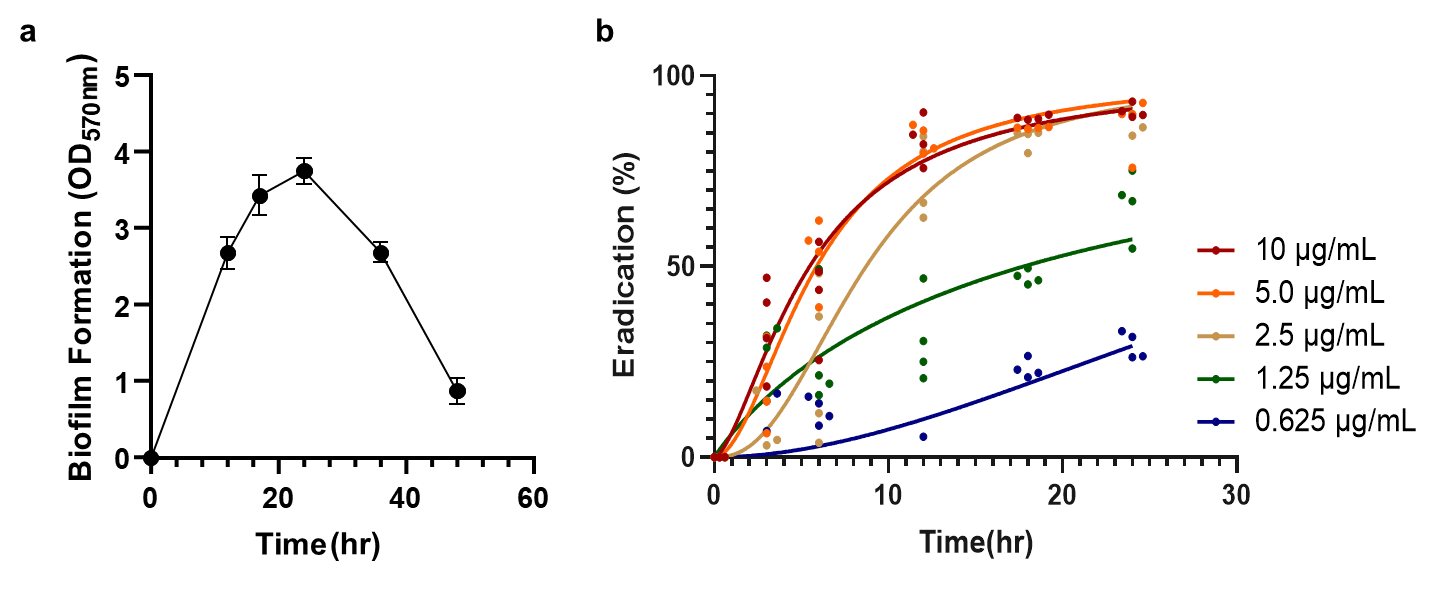


**Supplementary Figure S1. a,** Time course of biofilm formation of MRSA ATCC 43300 (n=3). **b,** Time course of biofilm eradication by various concentrations of elasnin (n=4).

**
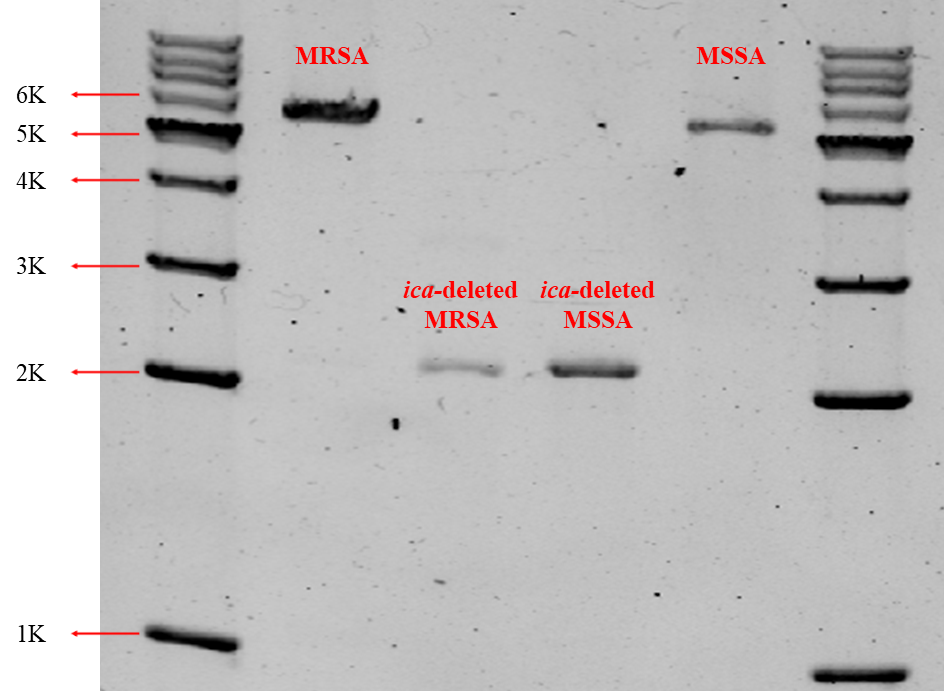
****Supplementary Figure S2.** PCR confirmation of *ica*-deleted mutants of MRSA ATCC 43300 and MSSA ATCC 25923. PCR was conducted with genomic DNA extracted using TIANamp Bacteria DNA Kit (Tiangen Biotech, Beijing, China) with the procedure described in the Method section. The total size of the amplified fragment from the wild-type genome is 5,575 bp and the size of *ica* operon is 3,416 bp. Presence of a band at ~2,100 bp (*ica*-deleted MRSA and MSSA) but not at ~5,500 bp (MRSA and MSSA) indicated successful deletion of *ica* operon (*ica*ADBC).

#
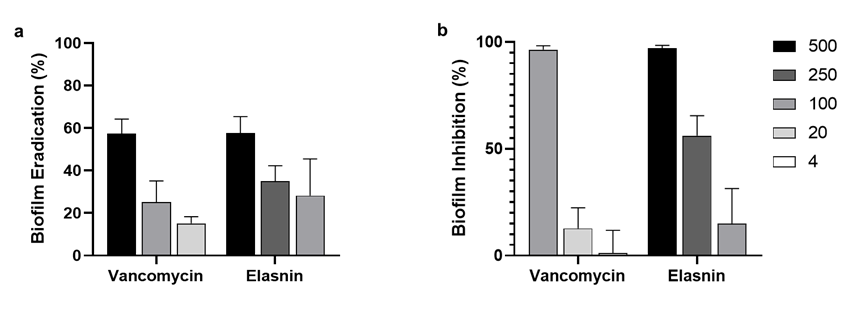


# Supplementary Figure S3. Antibiofilm activity of elasnin and vancomycin against MRSA in modified LCWB model. a, Minimum concentration needed to eradicate 50% of pre-formed biofilms of elasnin (n=6) and vancomycin (n=3) (average ± standard deviation). b, Minimum concentration needed to inhibit 90% of biofilm formation of elasnin (n=6) and vancomycin (n=3) (average ± standard deviation). Legend shows the concentration of vancomycin/elasnin in μg/mL.


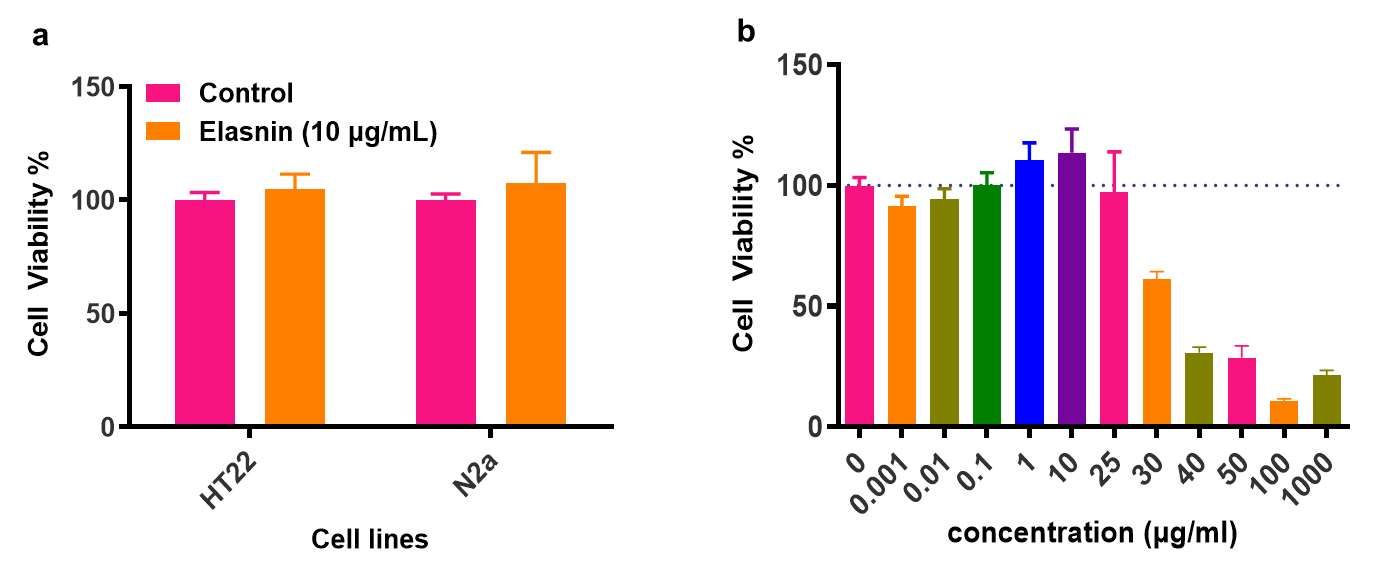


**Supplementary Figure S4**. Cytotoxicity of elasnin against nerve cells. **a,** Cell viability of HT22 and Neuro2a upon treatment with 10 μg/mL of elasnin for 24 hours (n=8). **b,** Cell viability of HT22 cells after treatment various concentrations of elasnin after 24 hours (n=6).


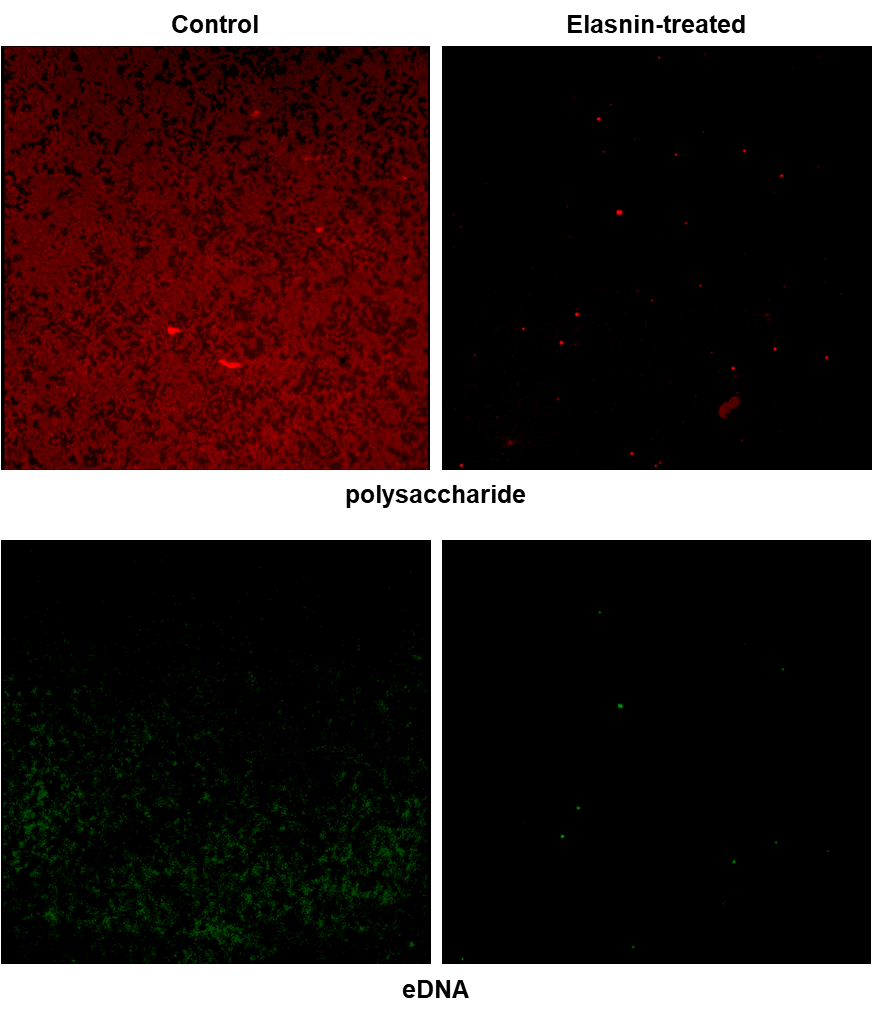


**Supplementary Figure S5**. Separate staining images of polysaccharides and eDNA within MRSA biofilms. The polysaccharide (top figures, red color) was stained using concanavalin A that selectively binds to a-mannopyranosyl and a-glucopyranosyl residues, while eDNA (bottom figures, green color) was stained with TOTO-1 that binds cell-impermeant nucleic acids. Images were acquired simultaneously using two channels and the experiment was performed in triplicates.


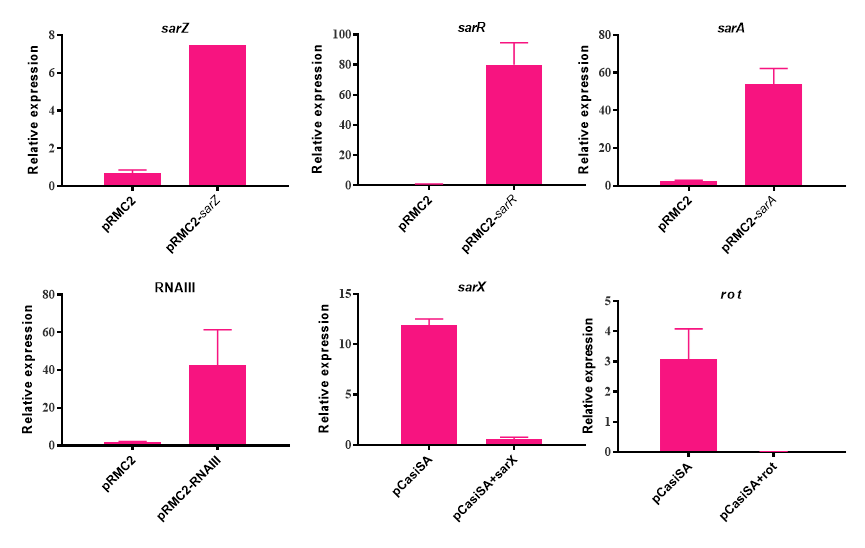


**Supplementary Figure S6**. Quantitative real-time PCR validation of gene expression in the mutants (n=6). Gene *sarZ*, *sarR*, *sarA* and RNA III was overexpressed using an expression vector pRMC2 while gene *sarX* and *rot* was transcriptionally inhibited by CRISPR/Cas9 transcription inhibition system pCasiSA. Relative expression levels of these genes were normalized to that of the reference gene *gyrB*.


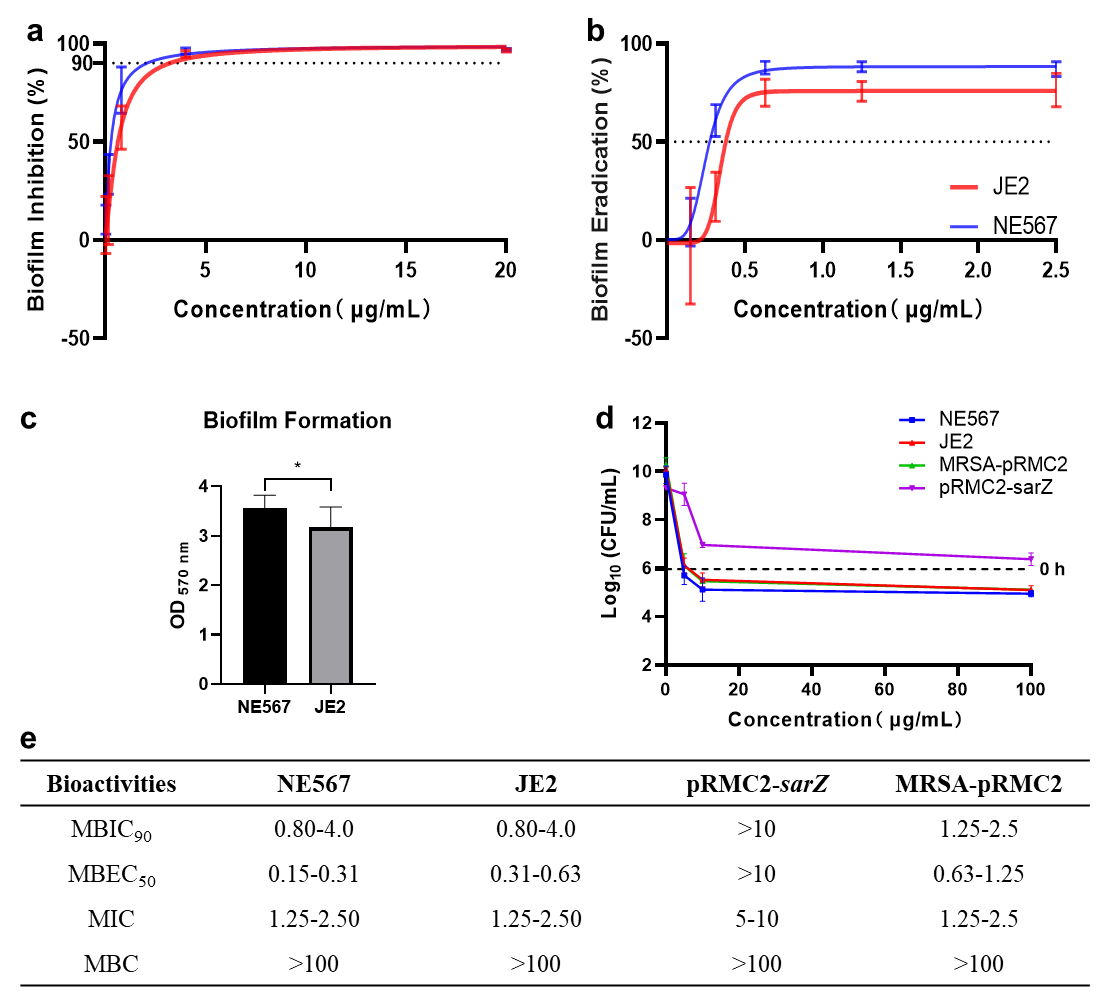


**Supplementary Figure S7**. Bioactivities of elasnin against *sarZ*-deleted MRSA mutant NE567, *sarZ*-overexpressed mutant pRMC2-*sarZ*, and their parent strain MRSA JE2 and MRSA-pRMC2. **a.** Minimum concentration needed to inhibit 90% of biofilm formation (n=12, average ± standard deviation). **b.** Minimum concentration needed to eradicate 50% of pre-formed biofilms (n=12, average ± standard deviation). **c**. Biofilm formation of *sarZ*-deleted MRSA mutant NE567 and its parent strain MRSA JE2. **d**. Cell viability of MRSA strains after 24 h of treatment with various concentrations of elasnin (*n*=3). The average inoculum size of all strains is ~10^6^ CFU/mL. **e.** Summary of the bioactivities.


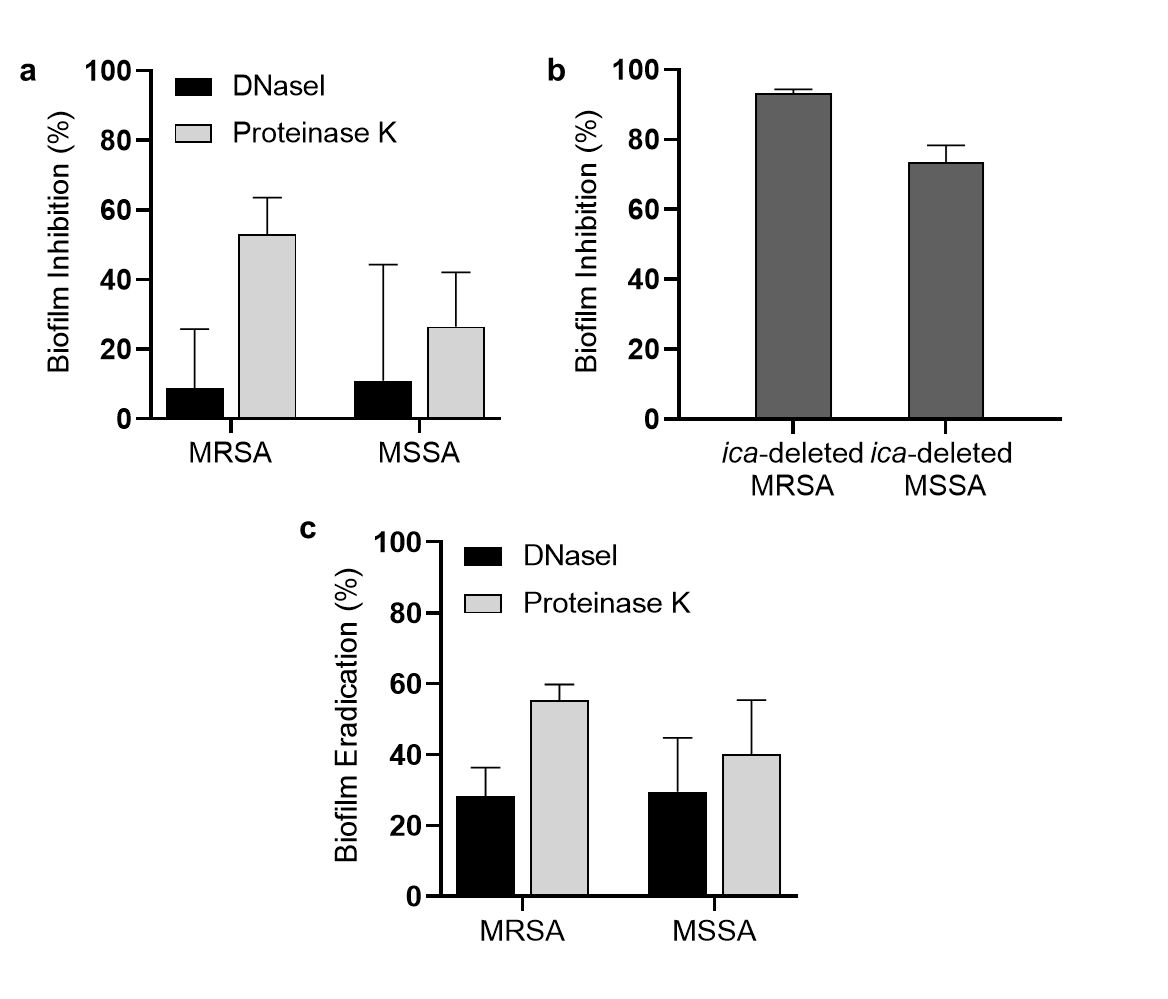


**Supplementary Figure S8**. Biochemical composition study on biofilms of MRSA and MSSA. **a,** Treatment of proteinase K resulted in a significant reduction in the biofilms, while treatment of DNaseI resulted in a mild reduction in the biofilms on both MRSA and MSSA. **b,** *ica*-deleted mutants of MRSA and MSSA shows significant reduction in biofilm formation (>70% biofilm inhibition compared to the wild-type). Biofilm inhibition of *ica*-deleted mutants was calculated based on the biofilm formation of the mutants compared to the biofilm formation of the wild-type with an empty plasmid (pCasSA). **c**, Treatment of DNase I and proteinase K caused detachment of established biofilms in both MRSA and MSSA.

# Supplementary Tables

| **Supplementary Table S1**. Bioassay results of the crude extracts from 12 actinobacterial strains. | | | | |
| --- | --- | --- | --- | --- |
| **Strain** | **Bioassay** ^a^ | | | Identification |
|  | **MIC**  **(****μg/mL)** | **MBIC (μg/mL)** | **MBEC (μg/mL)** |  |
| *Streptomyces mobaraensis*  DSM 40847 | < 4 | < 4 | < 4 | Elasnin |
| *Nocardiopsis potens*  DSM 45234 | < 4 | > 100 | > 100 | Xanthone |
| *Streptomyces scabrisporus*  DSM 41855 | < 4 | > 100 | > 100 | Hitachimycin |
| *Streptomyces sulphureus*  DSM 40104 | 20-100 | > 100 | > 100 | Resistomycin |
| *Kutzneria albida*  DSM 43870 | 20-100 | > 100 | > 100 | - |
| *Streptomyces fulvissimus*  DSM 40593 | > 100 | > 100 | > 100 | - |
| *Streptomyces exfoliates*  DSM 41693 | > 100 | > 100 | > 100 | - |
| *Saccharothrix espanaensis*  DSM 44229 | > 100 | > 100 | > 100 | - |
| *Nocardiopsis synnemataformans*  DSM 44143 | > 100 | > 100 | > 100 | - |
| *Sciscionella marina*  DSM 45152 | > 100 | > 100 | > 100 | - |
| *Nonomuraea coxensis*  DSM 45129 | > 100 | > 100 | > 100 | - |
| *Streptomyces cattleya*  DSM 46488 | > 100 | > 100 | > 100 | - |

| **Supplementary Table S2. Plasmids and bacterial strains used in this study.** | | |
| --- | --- | --- |
| **Strains/plasmids** | **Description** | **Reference/Source** |
| ***E. coli*** | | |
| DH5α | Host strain for plasmid | Invitrogen |
| Top10 | Host strain for plasmid | Invitrogen |
| ***S. aureus*** | | |
| ATCC25923 | Wild-type methicillin-sensitive *S. aureus* | ATCC |
| ATCC25923pCasSA | ATCC25921 with pCasSA | This study |
| ATCC25923Δ*ica* | ATCC25921 with deletion of *ica* | This study |
| **MRSA** | | |
| ATCC43300 | Wild-type methicillin-resistant *S. aureus* | ATCC |
| ATCC43300pRMC2 | ATCC43300 with empty pRMC2 | This study |
| ATCC43300pRMC2-*sarA* | ATCC43300 with pRMC2-*sarA* | This study |
| ATCC43300pRMC2-*sarR* | ATCC43300 with pRMC2-*sarR* | This study |
| ATCC43300pRMC2-*sarZ* | ATCC43300 with pRMC2-*sarZ* | This study |
| ATCC43300pRMC2-RNAIII | ATCC43300 with pRMC2-RNAIII | This study |
| ATCC43300pCasiSA | ATCC43300 with empty pCasiSA | This study |
| ATCC43300pCasiSA-*rot* | ATCC43300 with pCasiSA-*rot* | This study |
| ATCC43300pCasiSA-*sarX* | ATCC43300 with pCasiSA-*sarX* | This study |
| ATCC43300pCasSA | ATCC43300 with pCasSA | This study |
| ATCC43300Δ*ica* | ATCC43300 with deletion of *ica* | This study |
| NR-47110 | *sarZ*-deleted mutant of *S. aureus subsp. aureus*, strain JE2 | NARSA |
| NR-46543 | *Staphylococcus aureus* (*S. aureus*) subsp. aureus, strain JE2 | NARSA |
| **Plasmids** | | |
| pRMC2 | Tetracycline-inducible expression vector | Addgene |
| pRMC2-*sarA* | pRMC2 with *sarA* | This study |
| pRMC2-*sarR* | pRMC3 with *sarR* | This study |
| pRMC2-*sarZ* | pRMC4 with *sarZ* | This study |
| pRMC2-RNAIII | pRMC5 with RNAIII | This study |
| pCasiSA | transcription inhibition vector, Km^r^, Cm^r^ | This study |
| pCasiSA-*rot* | pCasiSA with *rot* spacer | This study |
| pCasiSA-*sarX* | pCasiSA with *sarX* spacer | This study |
| pCasSA | S. aureus genome editing vector, Km^r^, Cm^r^ | Addgene |
| pCasSA-*icaA* | pCasSA with *icaA* spacer | This study |
| pCasSA-*icaD* | pCasSA with *icaD* spacer | This study |
| pCasSA-*ica*-MR | pCasSA derivative for *ica* deletion in MRSA | This study |
| pCasSA-*ica*-MS | pCasSA derivative for *ica* deletion in MSSA | This study |

| **Supplementary Table S3. Primers used in this study.** | | |
| --- | --- | --- |
| **Name** | **Sequence** | **Description** |
| pCasiF1 | CATGGATAAGAAATACTCAATAGGCTTAgctATCGGCA CAAATAGCGTCGG | pCasiSA construction |
| pCasiR1 | GTCTTTAAGGAAACTTTGTGGAACAATggcATCGACA TCATAATCACTTAAACG |  |
| pCasiF2 | CGTTTAAGTGATTATGATGTCGATgccATTGTTCCACA AAGTTTCCTTAAAGAC |  |
| pCasiR2 | cgattatgtcttttgcgcagtc |  |
| pCasiF3 | cgcaccagcgaaaactggt |  |
| pCasiR3 | CCGACGCTATTTGTGCCGATagcTAAGCCTATTGAGT ATTTCTTATCCATG |  |
| pcasiSA-checkF | agaaaggcggacaggtatcc | For checking/ confirmation |
| pcasiSA-checkR | ccgatagctaagcctattgag |  |
| ica-upF | tttgagatctgtccatacccatggTCTAGActactttaggaacgccac | *ica* gene deletion |
| ica-upR | acgaaaggtaggtaaagaaattattaagctatgttaaaaacacg |  |
| ica-downF | cgtgtttttaacatagcttaataatttctttacctacctttcgt |  |
| ica-downR | AAGATACAGGTATATTTTTCTGACTCGAGatcacataggcgcttatcaa |  |
| *ica*-spacerF1 | GAAAaagcgaagtcagacacttgc |  |
| *ica*-spacerR1 | AAACgcaagtgtctgacttcgctt |  |
| *ica*-spacerF2 | GAAAtcgctatatcgtgtgtcttt |  |
| *ica*-spacerR2 | AAACaaagacacacgatatagcga |  |
| pCasSA-checkF | gttcctggccttttgctggccttt |  |
| pCasSA-checkR | tccgacgctatttgtgccgatatc |  |
| *rot*-spacer-F1 | GAAAgttgacgatgaaagaacagt | *rot* spacer for pCasiSA |
| *rot*-spacer-R1 | AAACactgttctttcatcgtcaac |  |
| *rot*-spacer-F2 | GAAAagattaacgcagttactacg |  |
| *rot*-spacer-R2 | AAACtacgttctagtacgcttata |  |
| *sarX*-spacer-F1 | GAAAagcaatgtttctaatttctc | *sarX* spacer for pCasiSA |
| *sarX*-spacer-R1 | AAACgagaaattagaaacattgct |  |
| *sarA*-sacI-F | CGAGCTCaatagggaggttttaacc | Amplification of *sarA* fragment |
| *sarA*-kpnI-R | GGTACCCCttatagttcaatttcgt |  |
| *sarA*-kpnI-R1 | GGTACCttatagttcaatttcgt |  |
| RNAIII-sacI-F | CGAGCTCatcacagagatgtgatggaaaatagttg | Amplification of RNAIII fragment |
| RNAIII-kpnI-R | GGTACCCCaaaaaaggccgcgagctt |  |
| RNAIII-kpnI-R1 | GGTACCaaaaaaggccgcgagctt |  |
| *sarR*-sacI-F | CGAGCTCttgctacaacaagatgtgc | Amplification of *sarR* fragment |
| *sarR*-kpnI-R | GGTACCCCtcgttcaatgttattaaacgc |  |
| *sarR*-kpnI-R1 | GGTACCtcgttcaatgttattaaacgc |  |
| *sarZ*-sacI-F | CGAGCTCggagtgattggatgtatgtag | Amplification of *sarZ* fragment |
| *sarZ*-kpnI-R | GGTACCCCcccatcatcttatgataggga |  |
| *sarZ*-kpnI-R1 | GGTACCcccatcatcttatgataggga |  |
| pRMC2-F | aatcctgttaccagtggctg | For checking/ confirmation |
| pRMC2-R | gatacctgtccgcctttctc |  |
| pRMC2-Tet-R | ggcgagtttacgggttgtta |  |
| *sarA*-rt-F | ggtcacttatgctgacaaat | qRT-PCR |
| *sarA*-rt-R | gctttaacaacttgtggttg |  |
| *sarR*-rt-F | agtcaacgcaacatttcaag |  |
| *sarR*-rt-R | aaggtttgaactctgagcac |  |
| *sarZ*-rt-F | tcgaacacgtgaagagaaag |  |
| *sarZ*-rt-R | ctgatgcttctcgttctgaa |  |
| *sarX*-rt-F | tagaaacattgcttggcttc |  |
| *sarX*-rt-R | atctagctcatccattgcag |  |
| RNAIII-rt-F | aatggattatcgacacagtg |  |
| RNAIII-rt-R | agcaaatgttactcacttgc |  |
| *rot*-rt-F | ccgtataagcgtactagaac |  |
| *rot*-rt-R | cttgcaatcgcatcactgat |  |
| *gyrB*-rt-F | cgttaattgaagcaggctatgtg |  |
| *gyrB*-rt-R | tggtgttggattcaattcagatt |  |

| **Supplementary Table S4. MICs recorded during the 45-days resistance study.** | | | | | | | | | |
| --- | --- | --- | --- | --- | --- | --- | --- | --- | --- |
| **Day** | **MIC (range, μg/mL) ^a^** | | | **MIC (μg/mL) ^a^** | | | **Fold change in MIC ^b^** | | |
|  | **Ela** | **Van** | **Cip** | **Ela** | **Van** | **Cip** | **Ela** | **Van** | **Cip** |
| 1 | 2.5-5 | 0.625-1.25 | <0.125 | 5 | 1.25 | 0.125 | 1 | 1 | 1 |
| 2 | 5-10 | 0.625-1.25 | <0.125 | 10 | 1.25 | 0.125 | 2 | 1 | 1 |
| 3 | 2.5-5 | 5-10 | <0.125 | 5 | 10 | 0.125 | 1 | 8 | 1 |
| 4 | 1.25-2.5 | 0.625-1.25 | <0.125 | 2.5 | 1.25 | 0.125 | 0.5 | 1 | 1 |
| 5 | 5-10 | 0.625-1.25 | 0.25-0.5 | 10 | 1.25 | 0.5 | 2 | 1 | 4 |
| 6 | 2.5-5 | 1.25-2.5 | 0.25-0.5 | 5 | 2.5 | 0.5 | 1 | 2 | 4 |
| 7 | 1.25-2.5 | 0.625-1.25 | 0.25-0.5 | 2.5 | 1.25 | 0.5 | 0.5 | 1 | 4 |
| 8 | 1.25-2.5 | 1.25-2.5 | 0.25-0.5 | 2.5 | 2.5 | 0.5 | 0.5 | 2 | 4 |
| 9 | 1.25-2.5 | 2.5-5 | 0.25-0.5 | 2.5 | 5 | 0.5 | 0.5 | 4 | 4 |
| 10 | 1.25-2.5 | 2.5-5 | 0.25-0.5 | 2.5 | 5 | 0.5 | 0.5 | 4 | 4 |
| 11 | 1.25-2.5 | 2.5-5 | 0.5-1 | 2.5 | 5 | 1 | 0.5 | 4 | 8 |
| 12 | 2.5-5 | 5-10 | 0.5-1 | 5 | 10 | 1 | 1 | 8 | 8 |
| 13 | 2.5-5 | 2.5-5 | 0.5-1 | 5 | 5 | 1 | 1 | 4 | 8 |
| 14 | 5-10 | 2.5-5 | 0.125-0.25 | 10 | 5 | 0.25 | 2 | 4 | 2 |
| 15 | 2.5-5 | 2.5-5 | 0.25-0.5 | 5 | 5 | 0.5 | 1 | 4 | 4 |
| 16 | 2.5-5 | 2.5-5 | 0.5-1 | 5 | 5 | 1 | 1 | 4 | 8 |
| 17 | 2.5-5 | 2.5-5 | 0.5-1 | 5 | 5 | 1 | 1 | 4 | 8 |
| 18 | 2.5-5 | 2.5-5 | 1-2 | 5 | 5 | 2 | 1 | 4 | 16 |
| 19 | 2.5-5 | 2.5-5 | 1-2 | 5 | 5 | 2 | 1 | 4 | 16 |
| 20 | 2.5-5 | 2.5-5 | 1-2 | 5 | 5 | 2 | 1 | 4 | 16 |
| 21 | 0.625-1.25 | 5-10 | 1-2 | 1.25 | 10 | 2 | 0.25 | 8 | 16 |
| 22 | 1.25-2.5 | 2.5-5 | 1-2 | 2.5 | 5 | 2 | 0.5 | 4 | 16 |
| 23 | 0.625-1.25 | 2.5-5 | 2-4 | 1.25 | 5 | 4 | 0.25 | 4 | 32 |
| 24 | 1.25-2.5 | 2.5-5 | 4-8 | 2.5 | 5 | 8 | 0.5 | 4 | 64 |
| 25 | 1.25-2.5 | 5-10 | 8-16 | 2.5 | 10 | 16 | 0.5 | 8 | 128 |
| 26 | 2.5-5 | 2.5-5 | 8-16 | 5 | 5 | 16 | 1 | 4 | 128 |
| 27 | 2.5-5 | 2.5-5 | 8-16 | 5 | 5 | 16 | 1 | 4 | 128 |
| 28 | 1.25-2.5 | 2.5-5 | 8-16 | 2.5 | 5 | 16 | 0.5 | 4 | 128 |
| 29 | 2.5-5 | 1.25-2.5 | 8-16 | 5 | 2.5 | 16 | 1 | 2 | 128 |
| 30 | 1.25-2.5 | 2.5-5 | 8-16 | 2.5 | 5 | 16 | 0.5 | 4 | 128 |
| 31 | 1.25-2.5 | 2.5-5 | 8-16 | 2.5 | 5 | 16 | 0.5 | 4 | 128 |
| 32 | 2.5-5 | 1.25-2.5 | 8-16 | 5 | 2.5 | 16 | 1 | 2 | 128 |
| 33 | 1.25-2.5 | 2.5-5 | 8-16 | 2.5 | 5 | 16 | 0.5 | 4 | 128 |
| 34 | 1.25-2.5 | 2.5-5 | 8-16 | 2.5 | 5 | 16 | 0.5 | 4 | 128 |
| 35 | 1.25-2.5 | 2.5-5 | 8-16 | 2.5 | 5 | 16 | 0.5 | 4 | 128 |
| 36 | 1.25-2.5 | 2.5-5 | 16-32 | 2.5 | 5 | 32 | 0.5 | 4 | 256 |
| 37 | 1.25-2.5 | 2.5-5 | 16-32 | 2.5 | 5 | 32 | 0.5 | 4 | 256 |
| 38 | 1.25-2.5 | 2.5-5 | 16-32 | 2.5 | 5 | 32 | 0.5 | 4 | 256 |
| 39 | 1.25-2.5 | 2.5-5 | 16-32 | 2.5 | 5 | 32 | 0.5 | 4 | 256 |
| 40 | 1.25-2.5 | 2.5-5 | 16-32 | 2.5 | 5 | 32 | 0.5 | 4 | 256 |
| 41 | 1.25-2.5 | 2.5-5 | 16-32 | 2.5 | 5 | 32 | 0.5 | 4 | 256 |
| 42 | 1.25-2.5 | 2.5-5 | 16-32 | 2.5 | 5 | 32 | 0.5 | 4 | 256 |
| 43 | 1.25-2.5 | 1.25-2.5 | 16-32 | 2.5 | 2.5 | 32 | 0.5 | 2 | 256 |
| 44 | 1.25-2.5 | 2.5-5 | 16-32 | 2.5 | 5 | 32 | 0.5 | 4 | 256 |
| 45 | 1.25-2.5 | 2.5-5 | 16-32 | 2.5 | 5 | 32 | 0.5 | 4 | 256 |
| **^a^** Experiment was performed in triplicates and the MIC was recorded as the highest concentrations among all replicates on each day.  **^b^** The fold change is the ratio of the MICs at any other day compared to the one recorded at the first day. | | | | | | | | | |

| **Supplementary Table S5. Biofilm inhibiting activities of elasnin against mutants.** | | | |
| --- | --- | --- | --- |
| **Strain** | **MBIC_90_** | **Biofilm Inhibition**  **(5 μg/mL, %)** | **Biofilm formation (OD_570_)** |
| Wild Type | 1.25-2.5 | 96.91±3.72 | 1.96±0.79 |
| pRMC2 | 1.25-2.5 | 96.60±1.25 | 2.48±0.54 |
| pRMC2+*sarA* | 5-10 | 72.17±5.20 | 1.86±0.29 |
| pRMC2+*sarR* | >10 | 30.22±9.67 | 0.46±0.10 |
| pRMC2+RNAIII | >10 | 18.32±9.65 | 0.15±0.03 |
| pRMC2+*sarZ* | >10 | -10.44±11.44 | 0.46±0.06 |
| pCasiSA | 2.5-5.0 | 90.96±1.40 | 2.18±0.44 |
| pCasiSA+*rot* | 5-10 | 75.66±4.25 | 1.43±0.44 |
| pCasiSA+sarX | 5-10 | 40.56±18.58 | 0.98±0.17 |

| **Supplementary Table S6. Biofilm inhibiting/eradicating efficiency of different antibiofilm agents against two *S. aureus* strains.** | | | | |
| --- | --- | --- | --- | --- |
|  | MRSA | | MSSA | |
|  | Inhibition | Eradication | Inhibition | Eradication |
| Elasnin | 99.01 ± 1.63 | 83.42 ± 8.26 | 98.86± 0.73 | 82.99 ± 3.76 |
| DNase I | 8.89 ± 16.94 | 28.14 ± 8.14 | 10.97 ± 33.34 | 29.50 ± 15.20 |
| Proteinase K | 53.15 ± 10.36 | 55.26 ± 4.50 | 26.47 ± 15.61 | 40.18 ± 15.18 |

The MBIC_90_ and MBEC_50_ of elasnin were same against MRSA ATCC43300 and MSSA ATCC 25923. The data shown above was the inhibition/eradication efficiency of elasnin (5 μg/mL), DNase I (100 U/mL), and proteinase K (100 μg/mL). The inhibition/eradication ratio (%) was presented as average ± standard deviation.

| **Supplementary Table S7. Antibiofilm activity of elasnin against two *S. aureus* strains.** | | | | |
| --- | --- | --- | --- | --- |
| Strains | Inhibition | | Eradication | |
|  | MBIC_90_ (μg/mL) | Efficiency | MBEC_50_ (μg/mL) | Efficiency |
| MRSA | 2.5 | 91.45 ± 7.69 | 1.25 | 65.52 ± 9.4 |
| MSSA | 2.5 | 83.76 ± 7.43 | 1.25 | 59.97 ± 8.88 |
